# A Single‐Capillary Open Microfluidic Probe for Robust and Versatile Spatial Multi‐Omics Using Ambient Mass Spectrometry Imaging

**DOI:** 10.1002/advs.78657

**Published:** 2026-11-05

**Authors:** Xiangyu Zhu, Yunkai Li, Jianing Wang, Chengyi Xie, Lei Guo, Yanyan Chen, Thomas Ka‐Yam Lam, Shao‐Wen Shi, Kun Wang, Baoliang Chen, Zongwei Cai

**Affiliations:** ^1^ School of Marine Sciences Hainan University Haikou China; ^2^ Department of Chemistry Hong Kong Baptist University Hong Kong China; ^3^ Single‐Cell Proteomics Research Center, ZJU‐Hangzhou Global Scientific and Technological Innovation Center Zhejiang University Hangzhou China; ^4^ State Key Laboratory of Soil Pollution Control and Safety Department of Environmental Science Zhejiang University Hangzhou China; ^5^ School of Chemistry College of Science Eastern Institute of Technology Ningbo Zhejiang China

**Keywords:** ambient mass spectrometry, lipid isomer, lipidomics, metabolomics, native mass spectrometry, proteomics, spatial omics

## Abstract

High‐resolution spatial mapping of tissue molecular architecture—from metabolites and lipid isomers to native proteins—remains a central challenge for ambient mass spectrometry imaging (MSI). Existing methods typically rely on multi‐capillary, alignment‐sensitive liquid‐extraction probes, compromising robustness and compatibility with structure‐resolved spatial omics. We mitigate this trade‐off by introducing a monolithic, knot‐shaped open microfluidic probe (Knot‐probe) fabricated from a single capillary. Knot‐probe achieves 40 µm scan‐direction spatial resolution and acquisition throughput up to 0.16 mm s^−1^, yet can be fabricated in ∼30 min with standard laboratory tools and operated reliably for over one month. Without sacrificing conventional spatial lipidomics and metabolomics, Knot‐probe enables structure‐resolved spatial omics, delivering high‐resolution imaging of phosphatidylcholine *sn*‐positional isomers via tandem MS and, in a pneumatically‐assisted variant, native MSI of proteins and proteoforms with higher throughput and enhanced sensitivity than with probes lacking pneumatic assistance. These capabilities arise from the integrated knot geometry, which ensures a stable micro‐liquid junction for localized extraction with a minimized sampling footprint and efficient mass transfer, while eliminating intricate probe alignment for robust operation. Readily integrated with standard electrospray ionization MS, Knot‐probe bridges conventional ambient MSI and emerging structural spatial omics, offering a broadly accessible approach across diverse biological and chemical applications.

## Graphical abstract

This study reports a knot‐shaped single‐capillary open microfluidic probe (Knot‐probe) for robust and versatile spatial omics, enabling ambient mass spectrometry imaging with high spatial resolution, throughput, and structure‐resolved capability. Knot‐probe mitigates the trade‐off between analytical performance and operational robustness, providing an accessible route to spatial lipidomics, metabolomics, isomer‐resolved lipid imaging, and native mass spectrometry imaging of proteins and proteoforms.

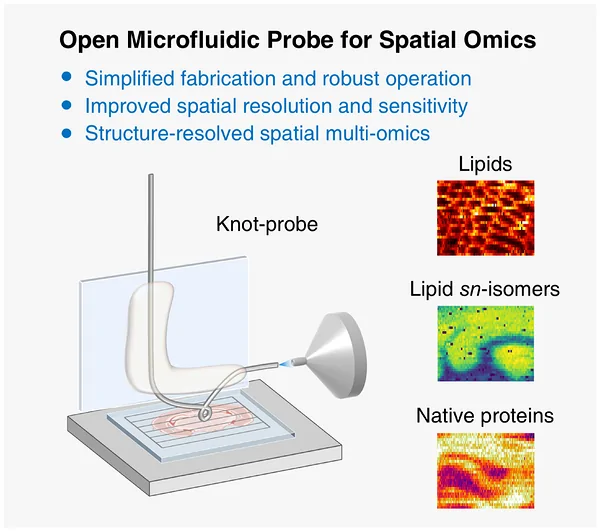

## 1 Introduction

Mass spectrometry imaging (MSI) is a transformative technology for understanding biology and disease, enabling label‐free visualization of hundreds of biomolecules directly from biological specimens [1, 2]. Powered by soft ionization techniques, MSI‐based spatial omics provide spatially resolved profiling of intact lipids, metabolites, and proteins, offering unprecedented insights into molecular organization and interactions within biological systems [3, 4]. Routine MSI‐based spatial omics workflows largely report sum composition information at the molecular‐formula level without resolving molecular structural features, such as structural diversities of lipid isomers or native protein conformations and proteoforms [5, 6]. These structural variants often exhibit distinct biosynthetic pathways, spatial distributions, and specific biological roles [7, 8]. Accordingly, there is a pressing need to integrate conventional formula‐based spatial omics with structure‐resolved spatial omics, linking molecular heterogeneity to structural information.

Matrix‐assisted laser desorption/ionization (MALDI) remains the dominant MSI platform due to its high throughput, sensitivity, and spatial resolution [9, 10]. Yet the need for matrix application and the use of denaturing conditions can limit access to certain structural details and native‐state proteomics. While advances such as on‐tissue derivatization [11], ion mobility spectrometry [12], and ion activation techniques [13, 14], improve structural characterization, flexible online chemistry and direct imaging of native proteins remain challenging in MALDI MSI. As a complementary approach, ambient liquid‐extraction MSI, such as desorption electrospray ionization (DESI) [15, 16] and nanospray DESI (nano‐DESI) [17], enable direct imaging of native biomolecules under atmospheric conditions with minimal sample preparation and flexible extraction solvent choices [18, 19]. For example, structural lipidomics methods can be readily adapted for imaging by integrating online reactions and ion activation with ambient MS [20, 21]; intact proteins can be imaged directly from tissues with their native folding retained using nondenaturing solvents [22, 23]. Realizing these advantages of ambient liquid‐extraction MSI for structure‐resolved spatial omics to be routinely adopted in practice, however, requires an optimized balance between these analytical capabilities and operational robustness.

The key to realize such multifunctionality of ambient liquid‐extraction MSI lies in the sampling probe, as its design critically determines spatial resolution, acquisition throughput, and operational robustness. Direct liquid‐extraction probes, exemplified by the nano‐DESI probe, provide high sensitivity by employing a capillary‐confined liquid junction at the probe−sample interface for continuous analyte extraction, maintained by probe tip geometry, interfacial wettability, and solvent flow rate [19]. To increase spatial resolution, common strategies include fine pulling and precise alignment of capillaries to minimize sampling footprint [24, 25, 26, 27], decreasing sample scan speed [28, 29], and employing oversampling [30, 31]. Achieving high spatial resolution, however, prolongs imaging time and reduces throughput, imposing stringent demands on probe robustness and accessibility. Integrated microfluidic probes that embed microchannels in a single microfabricated chip have recently been developed for surface sampling and ambient MSI [32, 33, 34, 35]. While elegant, these probes typically require expertise‐intensive microfabrication and assembly, hindering rapid design iteration and functional modification. As such, current probes often face a trade‐off between analytical performance and operational robustness: probes delivering high analytical performance are sophisticated to fabricate and operate, whereas more accessible probes offer limited multifunctionality. Mitigating this performance−robustness trade‐off is essential to fully exploit the unique advantages of ambient liquid‐extraction MSI for high‐quality, structure‐resolved spatial multi‐omics.

To mitigate this trade‐off, we drew inspirations from emerging open microfluidics systems that enable precise control of microscale liquids in open environments [36, 37, 38, 39]. Guided by these principles, we report a rationally‐designed single‐capillary open microfluidic probe with a knot‐shaped structure, termed Knot‐probe. This innovative knot geometry facilitates a stable, small liquid junction critical for high‐quality imaging while eliminating intricate probe alignment. The monolithic single‐capillary design simplifies fabrication (∼30 min), operation, and maintenance, with a practical in‐use lifespan exceeding one month. We validate the robust performance of the Knot‐probe through high‐spatial‐resolution and high‐throughput imaging of lipids and metabolites in tissue sections. Beyond conventional spatial omics, the Knot‐probe enables *sn*‐isomer‐resolved imaging of phosphatidylcholine (PC) via tandem MS and, notably, native MSI of proteins and proteoforms—two emerging capabilities central to structure‐resolved spatial omics with ambient MSI. By integrating the advantages of open microfluidics with a robust, straightforward design, the Knot‐probe offers an accessible route to ambient MSI for both conventional and advanced structural spatial omics across biological and chemical research.

## 2 Results and Discussion

### 2.1 Design of Knot‐Probe

The Knot‐probe is an integrated, compact device readily coupled to a mass spectrometer for ambient MSI (Figure 1, Figure S1). Analytes are continuously extracted from the sample sections through the Knot‐probe's open sampling port into the solvent and transferred to the spray emitter for electrospray ionization (Figure 1a). The fabrication is based on a single fused silica capillary shaped by a multi‐step flame‐bending and grinding process (Figure 1a, Figure S2, details provided in the Experimental Section). Four design features make the Knot‐probe a highly capable and robust ambient MSI setup for spatial omics (Figure 1b).

**FIGURE 1:**
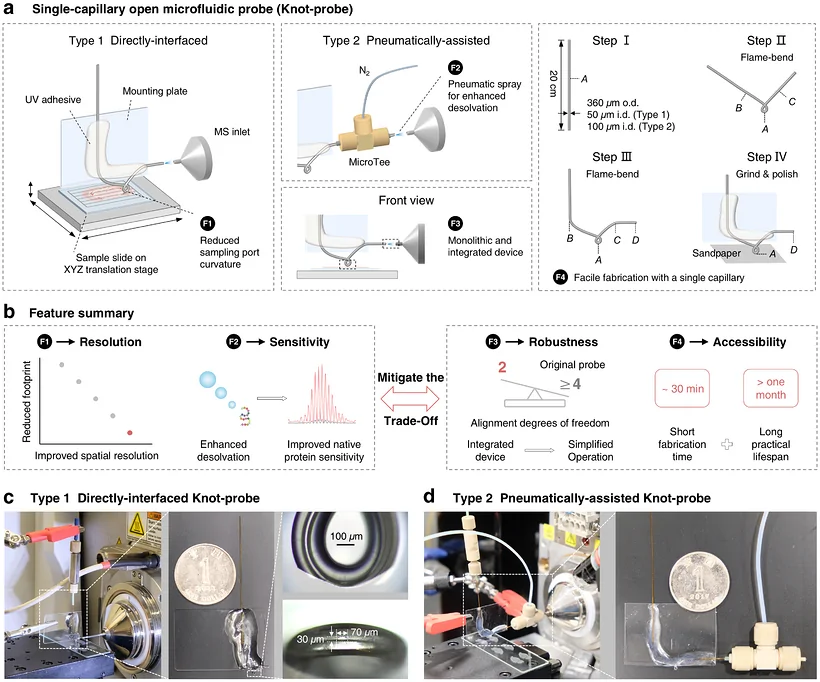
Knot‐shaped single‐capillary open microfluidic probe (Knot‐probe) for spatial multi‐omics using ambient mass spectrometry imaging (MSI). (a) Schematic illustration of Knot‐probe with two types. Illustrations are not to scale. (b) Summary of design features of Knot‐probe for robust and high‐performance ambient MSI. (c) Photograph of the Type 1 directly‐interfaced Knot‐probe, the microscopic view of the sampling port, and the interface to MS. (d) Photograph of the Type 2 pneumatically‐assisted Knot‐probe and the interface to MS.

First, the knot‐shaped geometry reduces the radius of curvature at the probe bottom end compared with previously reported U‐ or V‐shaped probes (Figure S3), yielding a smaller sampling‐port cross‐section and, consequently, a smaller liquid junction at the probe−sample interface. This reduced micro‐liquid junction facilitates high‐spatial‐resolution imaging with a smaller sampling footprint. Additionally, uniformly grinding the capillary's bottom surface at multiple orientations (described in the Experimental Section) ensures a near‐axisymmetric sampling port (Figure 1c), which improves micro‐liquid junction stability by minimizing asymmetric contact‐line pinning and suppresses lateral meniscus drift under shear during MSI experiments. Second, the Knot‐probe can interface directly with a mass spectrometer (Type 1, Figure 1c) for most applications, or be facilely modified with a pneumatic module (Type 2, Figure 1d, Figure S4) when used for native MSI of proteins. The pneumatically assisted variant enables enhanced electrospray desolvation, providing increased sensitivity for proteins. Third, the monolithic, single‐capillary design eliminates the need for precise positioning and alignment of different capillaries, enabling rapid, straightforward assembly and replacement of the MSI setup. Compared with previous alignment‐sensitive probes, establishing a stable liquid junction at the probe−sample interface for the Knot‐probe requires adjustment of only two distances using a micropositioner (Figure 1a): between the spray emitter and the MS inlet, and between the sampling port and the tissue section on a pre‐leveled translation stage. A stable liquid junction can be maintained across the imaging area of typical thin tissue sections without active feedback control of the sampling‐port‐to‐tissue distance. This reduced alignment degrees of freedom significantly simplifies operation and improves robustness. Lastly, the fabrication of the Knot‐probe does not require specialized microfabrication techniques; it can be completed in approximately 30 min with commonly accessible tools in MS laboratories, including fused silica capillary, butane lighter, and sandpaper (Figure 1a, Figure S2). Using a two‐step cleaning protocol (described in the Experimental Section), the Knot‐probe achieved a practical lifespan exceeding one month of intensive use without clogging or breakage. Three operators with routine MS experience were trained for the fabrication and operation of the Knot‐probe (Figure S5). The resulting total ion currents of line scans showed good reproducibility across the three experiments, demonstrating the accessibility of the Knot‐probe. Together, these four features corroborate that the Knot‐probe achieves a delicate optimization between the analytical performance and operational robustness. A comparison of the Knot‐probe and existing nano‐DESI probes summarizing their characteristics is provided in Table S1.

### 2.2 High‐Spatial‐Resolution Imaging of Lipids and Metabolites

We first validated the performance of the Knot‐probe via long‐time, high‐spatial‐resolution imaging of lipids (experimental parameters in Table S2). Three consecutive scans of mouse brain and testis sections, each lasting approximately 11 h, were performed spanning two days without probe clogging (Figure 2). Probe ionization stability was assessed by the fraction of pixels exhibiting signal dropout, which was below 3.7% in the selected ion images (Table S3). The full total ion current chromatogram and representative segments corresponding to Figure 2a are shown in Figure S6. The Knot‐probe provided high sensitivity, with an average signal‐to‐noise ratio (S/N) of 285 for all imaged lipid peaks within a single pixel (representative mass spectra provided in Figure S7). The overlay images of the reprojected uniform manifold approximation and projection (UMAP) dimensions of the MSI dataset and selected lipid ion images provided a clear delineation of the resolved histological features at high spatial resolution. In the cerebellum coronal section (Figure 2a), phosphatidylglycerol (PG) 34:1 and PG 44:12 (putatively assigned to *m*/*z* 747.5180 and 865.5018, respectively) rich in the granular layer, phosphatidylserine (PS) 36:2 (putatively assigned to *m*/*z* 786.5286) rich in the molecular layer, and sulfatide (SHexCer) 42:1;O3 (putatively assigned to *m*/*z* 906.6327) rich in the white matter were clearly differentiated. In the brain sagittal section (Figure 2b), similar differentiation of the cerebellar granular layer, molecular layer, and white matter was observed. The granule cell layer of the dentate gyrus and the pyramidal layer of cornu ammonis 3 in the hippocampus were also resolved as shown in the overlay image, with significantly enhanced phosphatidylinositol (PI) 38:4 signals (Figure 2b). Other than the mouse brain, the histological features of the mouse testis sections were resolved (Figure 2c). Complementary distributions of round‐shaped seminiferous tubules and polygon interstitial regions were observed, exemplified by PS 38:6 (putatively assigned to *m*/*z* 806.4972) and PG 44:10 (putatively assigned to *m*/*z* 869.5310), respectively. The signal intensity of PI 40:6 (putatively assigned to *m*/*z* 909.5488) showed a heterogeneous distribution across seminiferous tubules, suggesting a difference in their metabolic states. To provide an experimental evaluation of the spatial resolution achieved, representative line profiles exhibiting steep intensity gradient were calculated for the distance over which the signal increased from 20% to 80% of its maximum span (Figure 2d) [25, 40]. A spatial resolution of 40 µm along the scan axis was obtained under the specified experimental conditions, which is sufficiently high for routine ambient MSI with good robustness.

**FIGURE 2:**
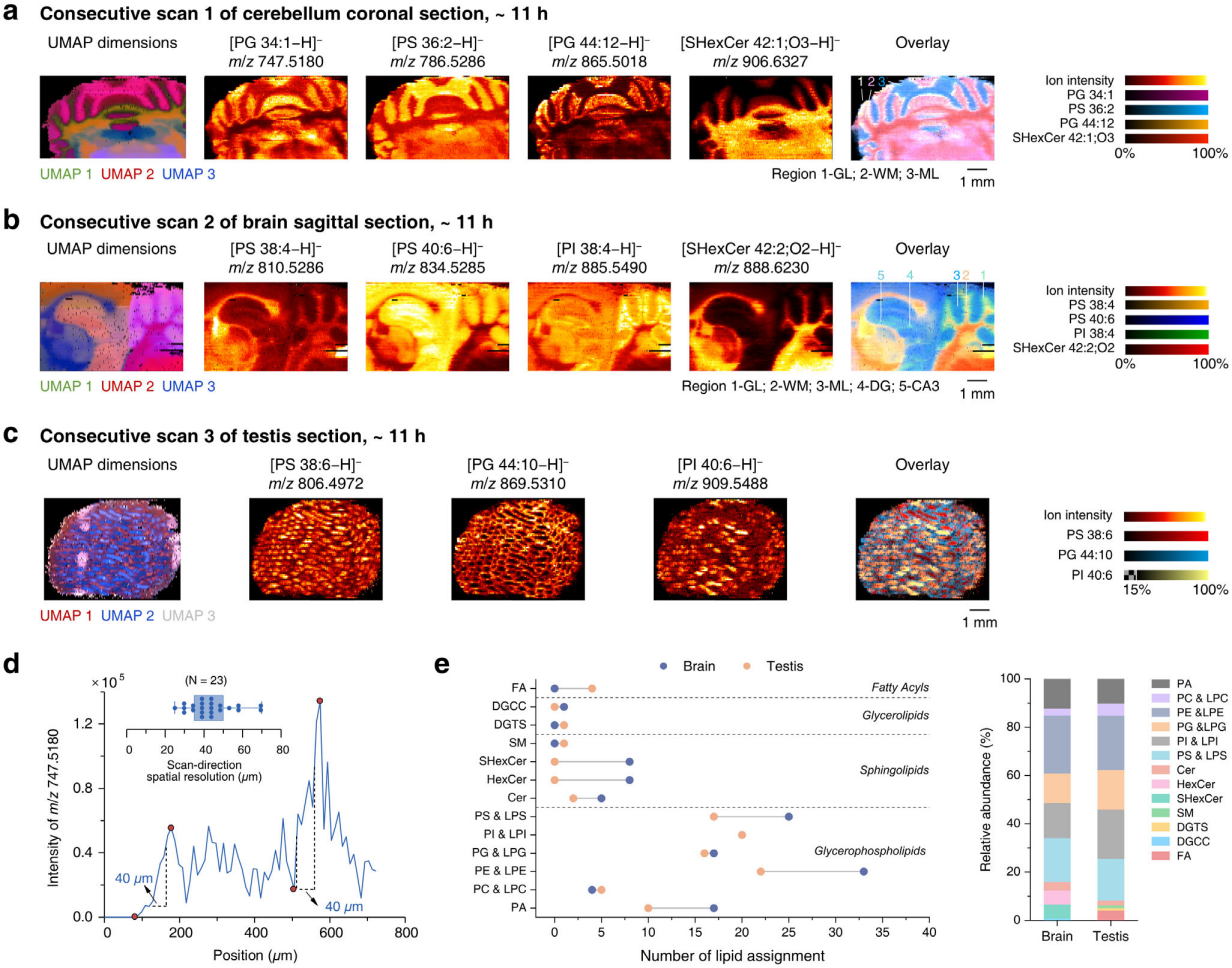
Performance validation of Knot‐probe via high‐spatial‐resolution imaging. Consecutive imaging of (a) a cerebellum coronal section, (b) a brain sagittal section, and (c) a testis section, each row showing the overlay image of reprojected uniform manifold approximation and projection (UMAP) dimensions, representative lipid ion images, and the overlay image of the selected ions. (d) Representative line profile of *m*/*z* 747.5180 for calculating scan‐direction spatial resolution achieved by the Knot‐probe. Dashed lines denote positions where the signal is at 20% and 80% of its span for the specific region indicated by red dots. Inset shows the scan‐direction spatial resolution calculated from 23 line profiles. (e) Comparison of assignments and relative abundance in lipid composition between mouse brain and testis sections. Sample scan speed, 37 µm s^−1^; vertical step size, 70 µm; in negative ionization mode. Lipid assignments are putative. Abbreviations: GL, granular layer; WM, white matter; ML, molecular layer; DG, dentate gyrus; CA, cornu ammonis. FA, fatty acids/esters; DGCC, diacylglycerylcarboxyhydroxymethylcholines; DGTS, diacylglyceryltrimethylhomoserines; SM; sphingomyelins; SHexCer, sulfatides; HexCer, hexosyl ceramides; Cer, ceramides; (L)PS, (lyso)phosphatidylserines; (L)PI, (lyso)phosphatidylinositols; (L)PG, (lyso)phosphatidylglycerols; (L)PE, (lyso)phosphatidylethanolamines; (L)PC, (lyso)phosphatidylcholines; PA, phosphatidic acids.

From the lipidomic perspective, the Knot‐probe enabled imaging of 138 and 98 lipids in the brain and testis sections, respectively, covering four lipid categories and 13 subclasses in negative ionization mode in the *m*/*z* range of 500−1000 (Figure 2e, Tables S4 and S5). This is comparable to the coverage reported for the conventional nano‐DESI probe and pulled flowprobe [26, 41]. Compositional differences in lipids were revealed in the two tissues (Figure 2e). The brain sections exhibited higher abundances of PS and lysoPS (LPS), phosphatidylethanolamines (PE) and LPE, phosphatidic acids (PA), SHexCer, and hexosyl ceramides (HexCer), while the testis sections exhibited higher abundances of (L)PI, (L)PG, and fatty acids (FA).

Representative high‐spatial‐resolution images of small‐molecule metabolites from mouse brain sagittal sections are shown in Figure S8. Taurine (putatively assigned to *m*/*z* 124.0074) showed high abundance across the section, while aspartic acid (putatively assigned to *m*/*z* 132.0302) and xanthine (putatively assigned to *m*/*z* 151.0264) were enhanced in the hindbrain, *N*‐acetyl‐aspartic acid (putatively assigned to *m*/*z* 174.0405) in the cerebellar molecular layer, and FA 20:1 (putatively assigned to *m*/*z* 309.2807) in the white matter. In total, 122 putatively assigned small‐molecule metabolites (*m*/*z* 100−500, excluding lipids) were imaged in negative ionization mode (Table S6). Collectively, we demonstrate the Knot‐probe's capability for robust, high‐spatial‐resolution MSI for conventional spatial lipidomics and metabolomics.

### 2.3 Compatibility of Knot‐Probe with High‐Throughput Acquisition

We next demonstrated Knot‐probe's operational robustness under different acquisition parameters (Figure 3, Table S2). By matching solvent flow rate to sample scan speed, imaging can be performed in either high‐spatial‐resolution or high‐throughput modes without changing probe configuration. At a scan speed of 160 µm s^−1^, corresponding to a 4.3‐fold increase in throughput (total acquisition time) relative to high‐spatial‐resolution imaging, a higher solvent flow rate (2 µL min^−1^) ensured faster analyte transfer from the sampling port to the MS inlet (Figure 3a). Ion images acquired at this scan speed showed no appreciable signal tailing (Figure 3b). We note that this throughput was obtained under the specified experimental conditions using the Orbitrap Fusion Tribrid; given that imaging throughput is ultimately constrained by the acquisition rate under these settings, mass spectrometers with higher acquisition rates should translate to higher imaging throughput [42].

**FIGURE 3:**
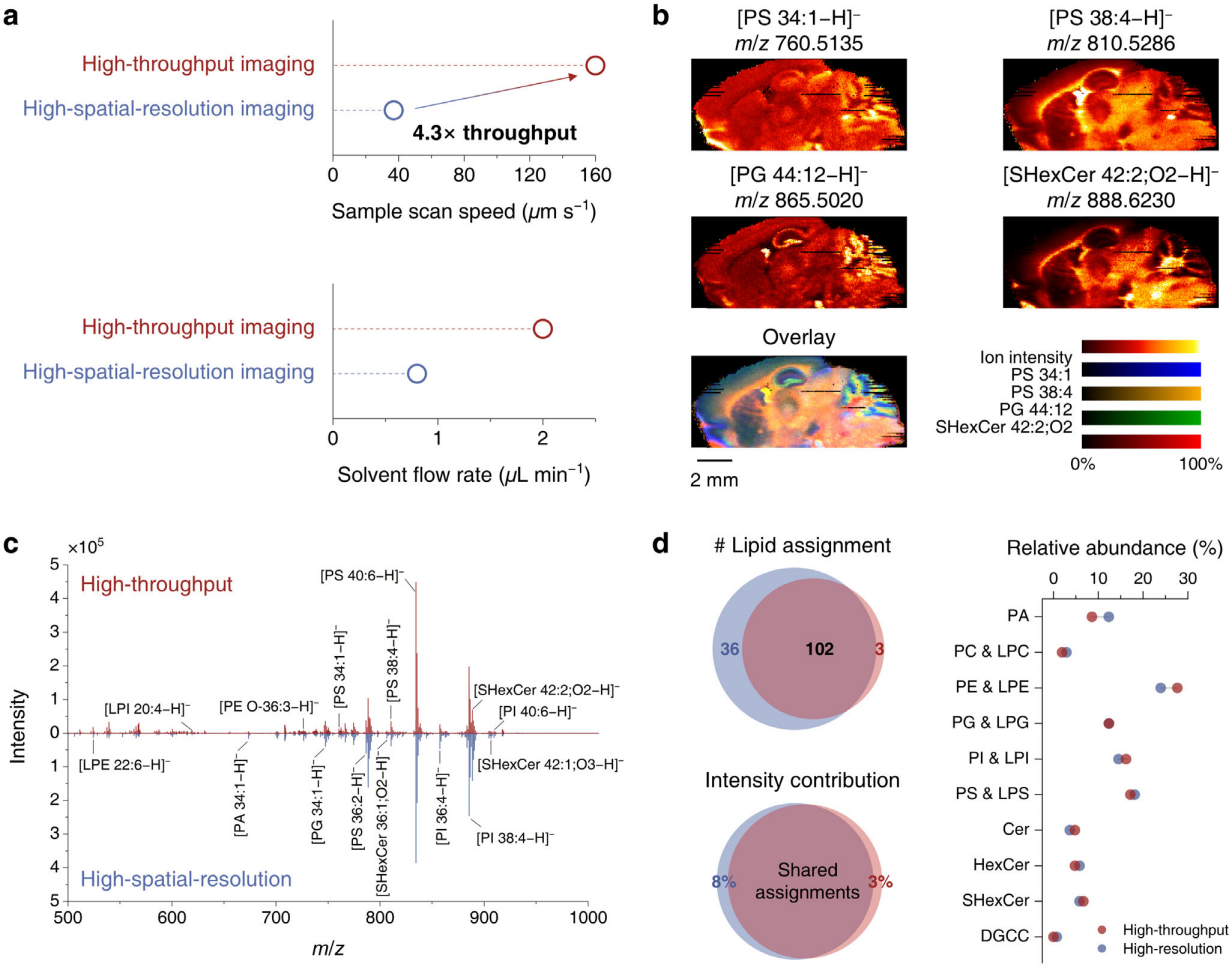
Compatibility of Knot‐probe with high‐throughput imaging. (a) Match in solvent flow rate and sample scan speed for high‐throughput and high‐spatial‐resolution acquisition. (b) Representative lipid ion images and the overlay image of the selected ions (*m*/*z* 760.5135, 810.5286, 865.5020, and 888.6230) of mouse brain sagittal sections. Sample scan speed, 0.16 mm s^−1^; vertical step size, 70 µm; in negative ionization mode. (c) Representative single‐pixel mass spectra of lipids acquired in high‐throughput and high‐spatial‐resolution experiments. (d) Comparison of lipid assignment numbers, intensity contribution, and relative compositional abundance between high‐throughput and high‐spatial‐resolution experiments. Lipid assignments are putative.

Despite the reduced number of pixels acquired at high scan speeds and the corresponding compromised ability to finely resolve histological details, spatial fidelity of biomolecules remained consistent with that of high‐spatial‐resolution imaging (Figures 3b and 2b). A similar spatial pattern was observed for PS 38:4 (putatively assigned to *m*/*z* 810.5286) and SHexCer 42:2;O2 (putatively assigned to *m*/*z* 888.6230) in the brain sagittal sections, with marked enrichment in the cerebellum white matter in both imaging modes. In the overlay image (Figure 3b), regions including the cerebellar granular layer, molecular layer, white matter, hippocampal dentate gyrus, cornu ammonis, and striatum were distinguished, confirming adequate spatial resolving power for high‐throughput imaging.

Altering sample scan speeds did not introduce substantial bias into lipid coverage, intensity, or overall compositional profile. The two acquisition modes shared highly similar representative single‐pixel mass spectra (Figure 3c) and comparable lipid coverage in mouse brain sections (Figure 3d, Table S4). A total of 138 and 105 lipids was putatively assigned in the high‐spatial‐resolution and high‐throughput experiments in negative ionization mode, respectively, with 102 shared assignments. These shared lipid assignments accounted for 92% and 97% of the total intensity of the assignments in the high‐spatial‐resolution and high‐throughput experiments, respectively. The relative abundances of major lipid classes were also consistent across the two acquisition modes (Figure 3d), indicating comparable overall lipid‐class composition.

Taken together, we establish the Knot‐probe as a capable, robust, and easily accessible tool through testing with conventional spatial lipidomics and metabolomics imaging experiments under varied scanning parameters. The combination of high spatial resolution, sensitivity, and chemical coverage provides a strong foundation to further develop the Knot‐probe for advanced ambient MSI applications, including high‐spatial‐resolution structural lipidomics and native proteomics.

### 2.4 *sn*‐Isomer‐Resolved Imaging of Phosphatidylcholines via Tandem MS

Spatial lipidomics increasingly demands high‐specificity methods that can resolve lipids with structural isomers [43]. We show the Knot‐probe for differentiating PC isomers with different fatty acyl *sn*‐positions on the glycerol backbone via tandem MSI at high spatial resolution (Figure 4). By doping the extraction solvent with ammonium bicarbonate, higher‐energy collisional dissociation (HCD) MS/MS of the bicarbonate adducts of PC produces *sn*‐1 specific fragment ions via radical‐directed fragmentation, and the ion intensities can be used to semi‐quantify *sn*‐positional isomers [20, 44].

**FIGURE 4:**
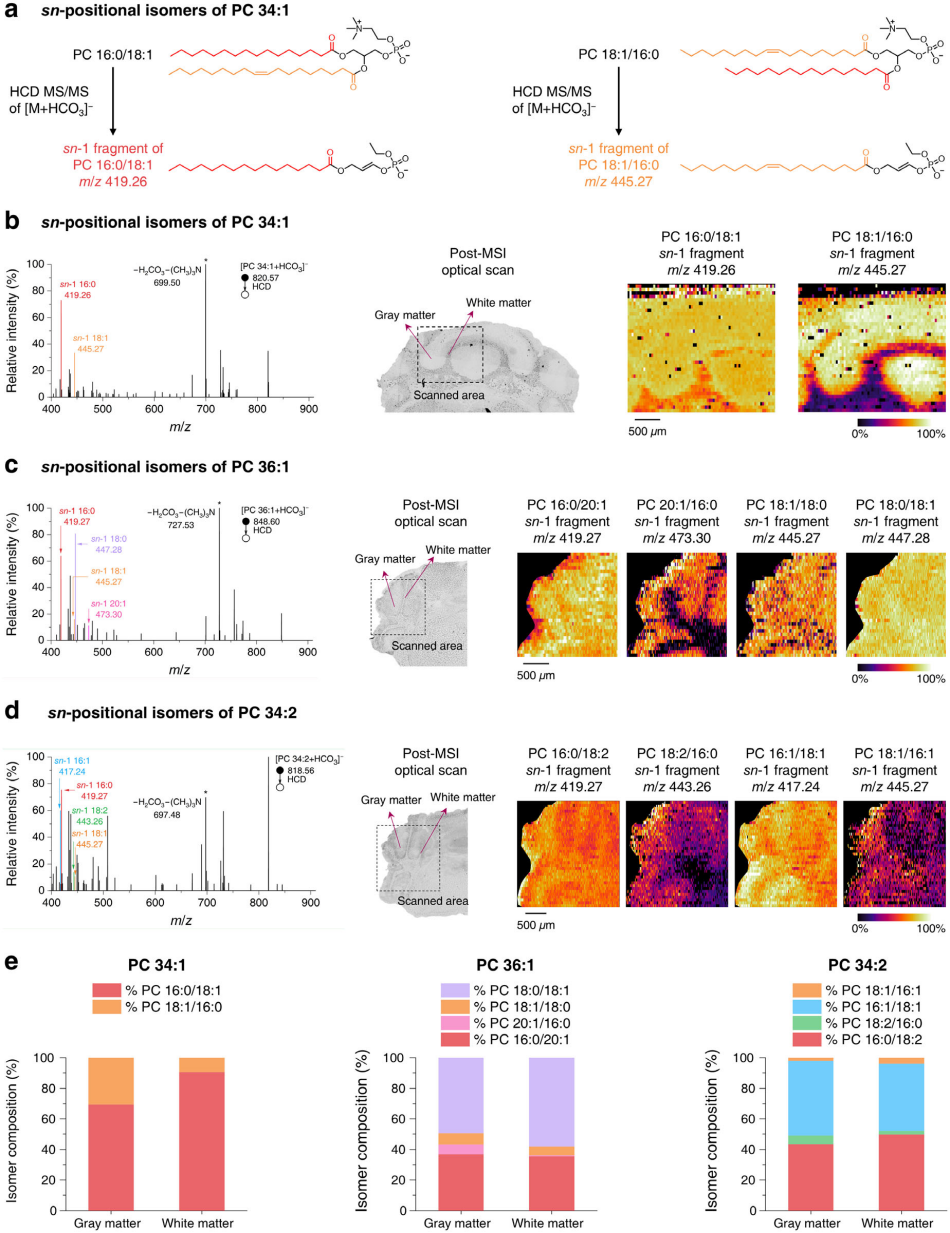
Distinguishing *sn*‐positional isomers of phosphatidylcholines via tandem MSI using Knot‐probe. (a) Schematic of higher‐energy collisional dissociation (HCD) MS/MS fragmentation of bicarbonate adducts of PC 16:0/18:1 and PC 18:1/16:0, yielding *sn*‐1 specific fragment ions. (b) Representative HCD MS/MS spectrum of [PC 34:1+HCO_3_]^−^ and imaging of *sn*‐1 specific fragment ions corresponding to isomers PC 16:0/18:1 and PC 18:1/16:0. (c) Representative HCD MS/MS spectrum of [PC 36:1+HCO_3_]^−^ and imaging of *sn*‐1 specific fragment ions corresponding to isomers PC 16:0/20:1, PC 20:1/16:0, PC 18:1/18:0, and PC 18:0/18:1. (d) Representative HCD MS/MS spectrum of [PC 34:2+HCO_3_]^−^ and imaging of *sn*‐1 specific fragment ions corresponding to isomers PC 16:0/18:2, PC 18:2/16:0, PC 16:1/18:1, and PC 18:1/16:1. (e) Root‐mean‐square‐normalized fragment ion intensity‐based isomer composition of PC in mouse brain sections. Sample scan speed, 66 µm s^−1^; vertical step size, 70 µm. Normalized collision energy, 28% for *m*/*z* 820.5 ± 0.5, 30% for *m*/*z* 848.6 ± 0.5 and 818.5 ± 0.5. Scanned sample areas are marked with dashed boxes in optical images of mouse brain coronal sections.

Taking PC 34:1 as an example, on‐tissue HCD MS/MS of [PC 34:1+HCO_3_]^−^ generated two fragment ions corresponding to the *sn*‐1 16:0 chain (*m*/*z* 419.26) and *sn*‐1 18:1 chain (*m*/*z* 445.27), together with *m*/*z* 699.50 ions indicative of neutral losses of H_2_CO_3_ and (CH_3_)_3_N (Figure 4a,b). The presence of two *sn*‐1 specific fragment ions indicated the coexistence of PC 16:0/18:1 and PC 18:1/16:0 isomers in the mouse brain coronal sections. Good fragment ion signals were obtained, with single‐pixel S/N up to 150. Tandem MSI revealed distinct distributions of the isomers in mouse cerebellum. Both PC 16:0/18:1 and PC 18:1/16:0 isomers were more abundant in the gray matter than the white matter indicated by their higher fragment ion intensities (Figure 4b). Another imaging experiment on a whole mouse cerebellum section further confirmed this distribution pattern (Figure S9), consistent with a previous study using a triple‐stage MS imaging method [21]. The difference in the abundance between the gray matter and the white matter was more pronounced for the PC 18:1/16:0 fragment ion (Figure 4b), suggesting a difference in metabolic specialization of the two isomers. Semi‐quantitative analysis based on root‐mean‐square‐normalized fragment ion intensities showed that the contribution of PC 18:1/16:0 to PC 16:0_18:1 isomers was higher in the gray matter, approximately 31%, than in the white matter, approximately 10% (Figure 4e), in line with previous observation using sequential collision‐induced dissociation and ozone‐induced dissociation [13].

In the case of PC 36:1, two pairs of isomers, namely PC 16:0_20:1 and PC 18:0_18:1, were imaged using their corresponding *sn*‐1 specific fragment ions (Figure 4c). PC 18:0/18:1 and PC 16:0/20:1 isomers with saturated *sn*‐1 acyl chains showed higher abundance (Figure 4e). The spatial distribution of PC 18:0/18:1 and PC 18:1/18:0 did not change substantially across the brain section, while the fragment ion of PC 20:1/16:0 showed markedly higher intensity in the gray matter (Figure 4c), in accordance with previous observation employing triple‐stage MS [21] and ion activation methods [13]. Isomer pairs of polyunsaturated PC were also differentiated. For PC 34:2, two pairs of PC 16:0_18:2 and PC 16:1_18:1 isomers were imaged using their corresponding *sn*‐1 specific fragment ions (Figure 4d), with PC 16:0/18:2 and PC 16:1/18:1 being the most abundant (Figure 4e). PC 16:0/18:2 and PC 18:2/16:0 were observed to slightly enrich in the white matter and gray matter, respectively, while both PC 16:1/18:1 and PC 18:1/16:1 were distributed relatively homogeneously across the brain section. Taken together, combining ambient liquid‐extraction MSI with bicarbonate adduct fragmentation, these results showcase the Knot‐probe's accessibility for structural spatial lipidomics applications requiring isomer‐resolved capabilities. An interesting extension to these capabilities would be the coupling of the Knot‐probe with advanced imaging acquisition approaches [45, 46] to improve the depth and coverage of structural annotation.

### 2.5 Native MSI of Proteins and Proteoforms Using Pneumatically‐Assisted Knot‐Probe

Native MS is an enabling technology in structural biology, which aims to preserve folded protein structures and noncovalent interactions in the gas phase [6, 47]. Ambient liquid‐extraction MS provides a gentle sampling environment well suited for native MSI of proteins directly from tissues. Being recently demonstrated [22, 23, 48], native ambient MSI using nondenaturing solvents has so far been constrained by inefficient aqueous‐spray desolvation, reduced sensitivity, and limited accessibility than denatured protein MSI [31, 49, 50, 51, 52]. We therefore adapted the Knot‐probe into a pneumatically assisted Type 2 configuration (Figure 1a,d, Figure S4) to test whether the same monolithic sampling geometry could support native protein MSI with useful sensitivity, spatial detail, and throughput.

Although Type 1 Knot‐probe demonstrated satisfactory performance for lipids and metabolites, it yielded little detectable native protein signals when using nondenaturing 200 mM ammonium acetate as the extraction solvent. Previous studies suggest that desolvation of protein‐containing droplets is critical for native protein ionization [53, 54]. Since aqueous solvents have high surface tension, we hypothesized that improving desolvation would lead to recovered protein ion signals. Incorporating a tee‐based pneumatic module provided gas assistance at the emitter (Figure 1a,d, Figure S4), which has been demonstrated effective for improving desolvation of aqueous solvents [55], and successfully recovered protein detection (Figure 5a). The ions exhibited narrow charge‐state distributions (4+ to 6+), indicating successful retainment of native‐like compact protein conformations under pneumatically‐assisted electrospray and appropriate source dissociation voltage (SDV). By contrast, control mass spectra acquired with 50% methanol/0.1% formic acid and 0 V SDV shifted to broader charge‐state envelopes (7+ to 13+), whereas 100 V SDV diminished protein signals (Figure S10). On‐tissue native top‐down MS/MS was performed to identify the detected proteins (Figure 5b). Specifically, the ion at *m*/*z* 1652.8 (6+) was identified as acyl‐CoA‐binding protein (ACBP). The detected proteoform carried N‐terminal methionine excision and acetylation and had a deconvoluted average mass of 9911 Da. The ions at *m*/*z* 1691.1 (5+) and *m*/*z* 1713.9 (5+) were identified as C‐terminal‐truncated ubiquitin (−GG) and polyubiquitin chain 1−76, respectively (Figures S11 and S12).

**FIGURE 5:**
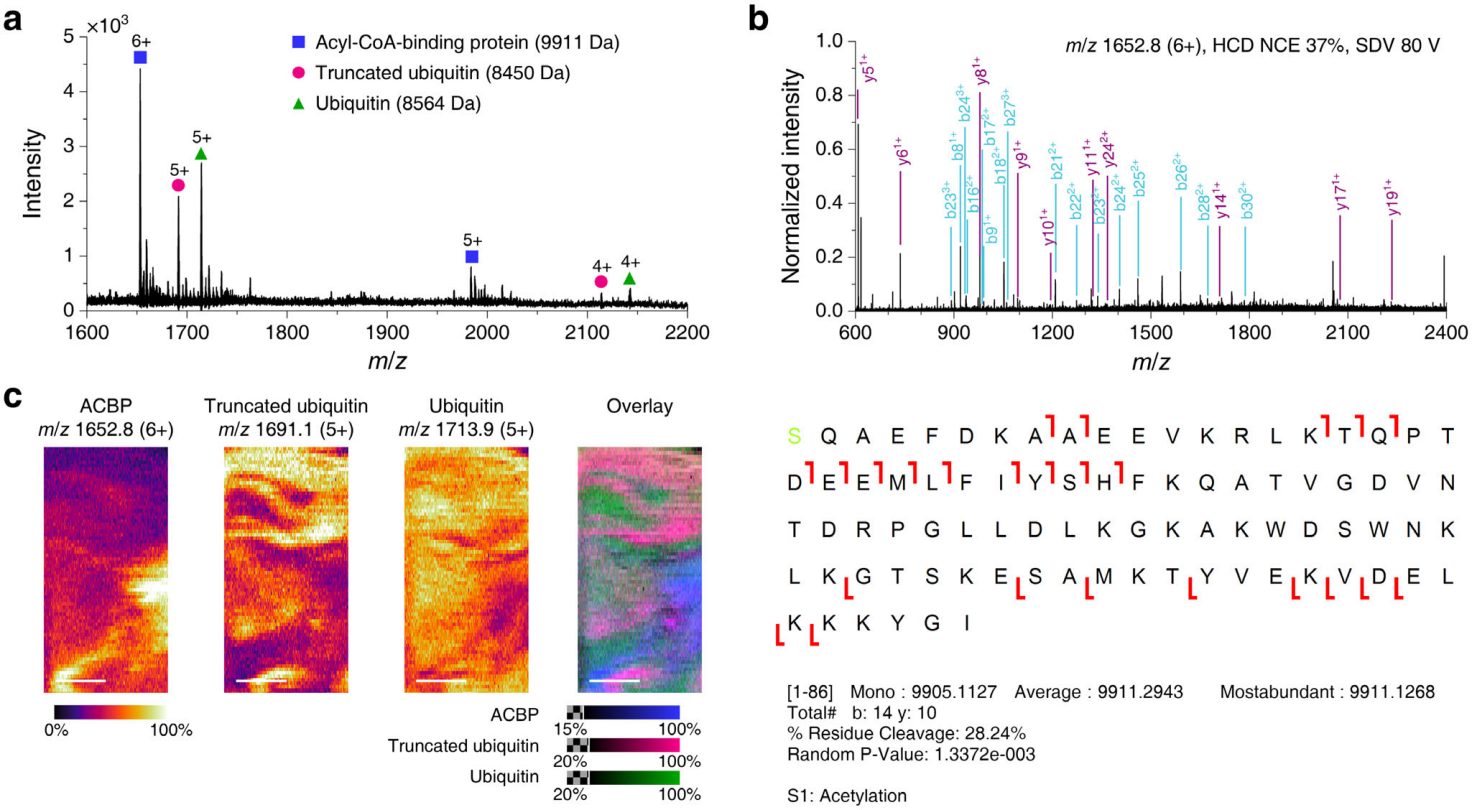
Native MSI of intact proteins in mouse brain sagittal sections using the pneumatically assisted Knot‐probe. (a) Representative native mass spectrum averaged over one line scan. (b) Native top‐down MS/MS spectra of the 6+ acyl‐CoA‐binding protein (ACBP) precursor ion *m*/*z* 1652.8 ± 2. (c) Ion images and overlay image of native ACBP (*m*/*z* 1652.8, 6+), C‐terminal‐truncated ubiquitin (*m*/*z* 1691.1, 5+), and ubiquitin (polyubiquitin chain 1−76, *m*/*z* 1713.9, 5+). Sample scan speed, 26 µm s^−1^; vertical step size, 70 µm. Scale bar, 1 mm.

Figure 5c shows the spatial distributions of the three proteins in the mouse brain sagittal sections. Native MSI performed using the Knot‐probe generated clearly resolved, protein‐specific ion images. ACBP and truncated ubiquitin exhibited largely complementary spatial patterns, while ubiquitin was distributed more uniformly across the section. Specifically, truncated ubiquitin ion signal was more pronounced in the hippocampal dentate gyrus, whereas ACBP ion signal was more prominent in the thalamus. Although previous nano‐DESI and liquid extraction surface analysis (LESA) MSI studies have demonstrated spatial differentiation of ACBP, ubiquitin, and truncated ubiquitin [49, 56], the Knot‐probe provided substantially higher spatial details at the microstructure level, without requiring sophisticated fabrication or alignment of separate sampling capillaries. Moreover, relative to a recent high‐resolution nano‐DESI native MSI experiment that required a 3 µm s^−1^ scan speed [28], the Knot‐probe was operated at 26 µm s^−1^ in this study, corresponding to a much higher throughput under native‐imaging conditions. We note that the protein coverage can be dependent on adjustable front‐end ion optics settings, which would be the direction for future improvement. Even with this limitation, the pneumatically assisted Knot‐probe extends the single‐capillary platform from lipid and metabolite MSI to intact protein and proteoform imaging. These results underscore the Knot‐probe as a practical platform for advancing structure‐resolved spatial proteomics and establish native MSI as a more widely usable analytical tool for structural biology, drug discovery, and interdisciplinary research.

## 3 Conclusions

The Knot‐probe provides a high‐performance, robust, and easily accessible solution for ambient liquid‐extraction MSI. Compared with previous nano‐DESI probes, the Knot‐probe bypasses the sophisticated processes of capillary alignment and positioning, streamlining fabrication (approximately 30 min), operation, and maintenance, and achieving a practical in‐use lifespan exceeding one month. The Knot‐probe is multifunctional for conventional spatial lipidomics and metabolomics in both high‐spatial‐resolution and high‐throughput acquisition, as well as advanced structure‐resolved spatial omics, including tandem MSI of phosphatidylcholine isomers and native MSI of proteins and proteoforms. These results offer the prospect of achieving spatial multi‐omics with a single ambient MSI modality, which not only complements the chemical space not amenable to MALDI MSI but also reduces the reliance on instrument resources and sample compatibility. Since the Knot‐probe readily couples to ESI MS instrumentation, it holds promise for applications that integrate surface sampling, reaction screening, and spatial imaging in a single probe platform. Integrating the Knot‐probe with an active feedback system for probe−sample distance control could further extend its applicability to samples with uneven surface topography [57, 58]. Overall, the Knot‐probe broadens the reach of ambient MSI and offers a practical route toward wider adoption of both conventional and structure‐resolved spatial multi‐omics across diverse biological and chemical research.

## 4 Experimental Section

### 4.1 Chemicals and Reagents

LC‐MS‐grade methanol and acetonitrile were obtained from VWR International. Ultrapure water was produced with a Milli‐Q purification system (Millipore, Bedford, MA) and used throughout the experiments. Ammonium acetate (NH_4_OAc, ≥ 99.0%) and ammonium bicarbonate (NH_4_HCO_3_, ≥ 99.5%) were supplied by Sigma‐Aldrich (St. Louis, MO). Glass microscope slides (25 × 75 × 1 mm) were from Citotest (Jiangsu, China). Cyclic olefin copolymer (COC) sheets (200 × 200 × 0.3 mm) were purchased from Yuxin Plastics (Shenzhen, China), and polydimethylsiloxane (PDMS) membranes (200 × 150 × 0.6 mm) from Weisichuang Company (Hangzhou, China). UV‐curing adhesive was obtained from Ergo (Kisling, Switzerland). Polymicro flexible fused‐silica capillaries (360 µm o.d., 50 µm i.d.; and 360 µm o.d., 100 µm i.d.) were purchased from Molex (Thief River Falls, MN).

### 4.2 Animal Tissues

C57BL/6 mice were supplied by the Laboratory Animal Service Center of the Chinese University of Hong Kong. Animals were maintained at 22°C and 55% relative humidity under a 12 h light/dark cycle, with free access to food and water. All procedures followed the Hong Kong Baptist University (HKBU) guidelines for experimental animal use and were approved by the HKBU Committee on the Use of Human & Animal Subjects in Teaching and Research. Brain and testis tissues were snap‐frozen, embedded in ice‐cold saline, and sectioned at −20°C into 10 µm slices on a CryoStar NX70 Cryostat (Thermo Fisher Scientific, Walldorf, Germany). The sections were thaw‐mounted on glass slides and vacuum‐desiccated for 30 min before MSI analysis.

### 4.3 Fabrication, Assembly, and Maintenance of Knot‐Probe

The Knot‐probe has two designs (Figure 1a). In Type 1, the shaped capillary was coupled directly to the mass spectrometer; in Type 2, the same sampling geometry was integrated with a downstream pneumatic spray module. Both configurations were prepared by the four‐stage workflow summarized in Figure 1a, and photographs of detailed fabrication procedures are provided in Figure S2. Typical fabrication time of a Knot‐probe was approximately 30 min.

Fused‐silica capillaries (20 cm long; 360 µm o.d., 50 µm i.d. for Type 1 and 360 µm o.d., 100 µm i.d. for Type 2) were used as the starting material. Each capillary was heated 6 cm from one end with a butane lighter and curved into an approximately 1 mm knot‐shaped loop, designated as point *A* in Figure 1a. Then, two additional bends defined the probe orientation. The capillary was reheated 0.5 cm upstream of point *A* (point *B*) and bent perpendicular to the loop plane, then reheated 0.5 cm downstream of point *A* (point *C*) and bent horizontally. The emitter length was set by cutting the capillary downstream of point *C* at point *D*, using 0.5 cm for Type 1 and 4 cm for Type 2. Heating during shaping would remove the capillary coating from the sampling‐port and emitter regions, as shown in Figure 1c,d. Lastly, the shaped capillary was fixed to either a glass slide or a COC sheet with UV‐curing adhesive. The bottom of the loop at point *A* was then polished with 1000‐, 2500‐, and 4000‐grit sandpapers to open the sampling port. Initial grinding on 1000‐grit paper was carried out from both orientations. After the inner bore became visible, the capillary was rinsed with water and dried with N_2_ to remove loose particles and prevent debris from entering the lumen. Final polishing on 2500‐ and 4000‐grit papers was performed from four directions, producing a small oval aperture approximately 70 µm long and 30 µm wide (Figure 1c). The emitter at point *D* was flattened on 2500‐grit paper, and all grinding steps were inspected under a microscope.

For Type 2 assembly (Figure 1a,d), the spray emitter was inserted into a PEEK MicroTee (P‐890, IDEX Health & Science), which served as the N_2_ gas junction. The MicroTee through‐hole was drilled from 0.15 to 0.6 mm before assembly. The emitter passed through two tubing sleeves (IDEX Health & Science). The upstream side was sealed with a Teflon sleeve (1/16″ o.d., 360 µm i.d.) and a MicroFerrule (F‐132, IDEX Health & Science), whereas the downstream side was sealed with a PEEK sleeve (1/16″ o.d., 0.5 mm i.d.) and a fluororubber sealer. The emitter protruded approximately 1 mm beyond the downstream sleeve. Nitrogen was introduced through 1/16″ o.d., 1 mm i.d. tubing and sealed with fluororubber. A schematic illustration of the pneumatic module is provided in Figure S4.

Particulates introduced during port polishing or MSI operation were removed by a routine rinse‐and‐flush maintenance procedure. The sampling port and emitter were first immersed in methanol, sonicated for 10 s, removed from the solvent, and dried under N_2_. For through‐capillary flushing, the sampling aperture was gently sealed against a PDMS membrane and methanol was pushed through the capillary with a syringe, causing debris to exit through the emitter (Figure S2). Excessive force was avoided during contact with the PDMS membrane to prevent capillary damage. This maintenance procedure kept individual probes unclogged over repeated MSI experiments.

### 4.4 MSI Platform Based on the Knot‐Probe

The Knot‐probe MSI platform comprised a syringe pump (Legato 180, KD Scientific) fitted with a 1.0 or 5.0 mL Hamilton glass syringe, an XYZ translation stage (CXPF100‐80175, Zolix) driven by custom motion‐control software, a Zolix micropositioner for probe placement, and a Dino‐Lite AM7115 digital microscope (AnMo Electronics Corporation) for ion‐source monitoring (Figure S1). Extraction solvent was delivered to the Knot‐probe through a fused‐silica transfer capillary (360 µm o.d., 250 µm i.d.), and the electrospray voltage was applied through the syringe needle. The probe was positioned above the sample slide and aligned with the inlet of either a Q Exactive Focus Orbitrap MS or an Orbitrap Fusion Tribrid MS (Thermo Scientific). During setup, the emitter‐to‐inlet distance and the sampling‐port‐to‐tissue distance were adjusted until a small, stable liquid junction formed at the tissue surface and a steady MS signal was obtained. To minimize variation in the sampling‐port‐to‐tissue distance across the imaging area, the translation stage was first leveled using a spirit level. The translation stage was then moved to bring each of the four corners of the rectangular tissue imaging area into view, and this distance was checked at each corner using the digital microscope to ensure the variation was kept within 20 µm. Analytes extracted into this junction were transported to the emitter by electrospray‐induced suction, the MS inlet vacuum, and, for Type 2, the Bernoulli flow generated by the pneumatic spray.

### 4.5 MSI Experiments

MSI data were acquired by raster‐scanning the sample on the motorized XYZ translation stage using the Knot‐probe, with a 70 µm vertical step size between lines. Experimental parameters, including solvent flow rate, sample scan speed, spray voltage, automatic gain control (AGC) target, and maximum injection time, were optimized to ensure a stable liquid junction and electrospray MS signal for different imaging modes (high‐spatial‐resolution MSI, high‐throughput MSI, tandem MSI, native MSI) and analyte classes (lipids, metabolites, proteins). Full parameters for each MSI experiment are listed in Table S2.

With the Type 1 Knot‐probe, imaging of lipids and metabolites in mouse brain and testis sections was performed using 90% methanol as the extraction solvent. For high‐spatial‐resolution imaging, sample scan speed was 33−37 µm s^−1^ with a solvent flow rate of 0.8−1 µL min^−1^; for high‐throughput imaging, sample scan speed was 0.16 mm s^−1^ with a solvent flow rate of 2 µL min^−1^.

For tandem MSI of lipid *sn*‐positional isomers, mounted tissue sections were first washed by rinsing with chilled 50 mM NH_4_HCO_3_ for 30 s and then left air‐dried [20]. Imaging was performed using 90% acetonitrile with 5 mM NH_4_HCO_3_ and 0.5% NH_4_OH as the extraction solvent [20]. Sample scan speed was 33 or 66 µm s^−1^ with a solvent flow rate of 0.8 µL min^−1^. HCD MS/MS spectra were acquired at 28% or 30% NCE with a 1 Da isolation window.

Native MSI of proteins was conducted with the Type 2 Knot‐probe using 200 mM ammonium acetate as the extraction solvent; sample scan speed was 26 µm s^−1^ with a solvent flow rate of 2.5 µL min^−1^ and a nebulizing gas pressure of 50 psi. No tissue washing was performed prior to imaging. The Orbitrap Fusion Tribrid MS was run in high mass range and intact protein mode, with the ion routing multipole pressure set at 8 mTorr, S‐lens RF at 150%, and SDV at 100 V.

The tuning and calibration of the Q Exactive Focus Orbitrap MS and Orbitrap Fusion Tribrid MS were performed using Pierce^TM^ LTQ Velos ESI positive ion calibration solution and Pierce^TM^ ESI negative ion calibration solution (Thermo Scientific) for positive and negative ionization modes, respectively.

### 4.6 Data Processing and Analysis

For each MSI acquisition, the Thermo .raw file was first converted to mzML with MSConvert (v3.0,ProteoWizard)and then to imzML with MT imzML Converter (ng; v1.4.1, MassTech). The imzML datasets were inspected and visualized in SCiLS Lab MVS (2025a Premium 3D, Bruker Daltonics). Ion images were normalized by RMS intensity. UMAP‐based dimensionality reduction was carried out according to our previously reported workflow [59, 60].

Putative assignments of lipids and small‐molecule metabolites to peaks with signal‐to‐noise ratios (S/N) > 10 were performed by matching to the LIPID MAPS Structure Database and the Human Metabolome Database, respectively, using mass errors < 5 ppm (Tables S4−S6). [M−H]^−^ and [M+Cl]^−^ ions were considered in negative ionization mode; [M+Cl]^−^ assignments were manually validated by the presence and abundance ratio of their ^37^Cl isotopologue peaks.

For protein identification, on‐tissue native top‐down MS/MS was performed. Precursor ions were selected in the ion trap using a 4 Da isolation window and fragmented by HCD, with the NCE and SDV optimized for each protein (Figure 5b, Figures S11 and S12). Fragment ions were recorded in the Orbitrap over *m*/ *z* 400−2500 at a resolution of 60000 (*m*/*z* 200). The S‐lens RF, AGC target, and maximum injection time were set to 50%, 1 × 10^6^, and 500 ms, respectively. The resulting. raw files were processed with MASH Native software developed by the Ge group [61]. Deconvolution was conducted using the eTHRASH algorithm with default settings. Database searches were performed against the *Mus musculus* proteome from UniProt (UP000000589). Assignments of b‐ and y‐type fragment ions were manually inspected using a mass tolerance of 10 ppm.

## Author Contributions

**Xiangyu Zhu**: conceptualization, methodology, investigation, writing – original draft, visualization, formal analysis. **Yunkai Li**: formal analysis, investigation, methodology. **Jianing Wang**: conceptualization, writing – review and editing, methodology, formal analysis, project administration, supervision, resources. **Chengyi Xie**: formal analysis, writing – review and editing, investigation. **Lei Guo**: investigation, visualization. **Yanyan Chen**: formal analysis, writing – review and editing, investigation. **Thomas Ka‐Yam Lam**: writing – review and editing, formal analysis, investigation. **Shao‐Wen Shi**: writing – review and editing, investigation. **Kun Wang**: writing – review and editing. **Baoliang Chen**: writing – review and editing, resources. **Zongwei Cai**: formal analysis, funding acquisition, project administration, resources, writing – review and editing, supervision.

## Conflicts of Interest

The authors declare no conflicts of interest.

## Supporting information




**Supporting File**: advs78657‐sup‐0001‐SuppMat.pdf.

## Data Availability

The data that support the findings of this study are available from the corresponding author upon reasonable request.
