# Supplementary material for: A Single‐Capillary Open Microfluidic Probe for Robust and Versatile Spatial Multi‐Omics Using Ambient Mass Spectrometry Imaging
Source: Adv Sci (Weinh). 2026 Nov 5:e78657. Online ahead of print. doi: 10.1002/advs.78657 (PMC13735432; doi:10.1002/advs.78657)
Supplement: Supplementary file 1 [file ADVS-9999-e78657-s001.pdf]

Supporting Information

A Single-Capillary Open Microfluidic Probe for Robust and Versatile Spatial Multi-Omics Using Ambient Mass Spectrometry Imaging

Xiangyu Zhu,[2] Yunkai Li,[2] Jianing Wang,[1]* Chengyi Xie,[2] Lei Guo,[2] Yanyan Chen,[2] Thomas Ka-Yam Lam,[2] Shao-Wen Shi,[3] Kun Wang,[4] Baoliang Chen,[4] Zongwei Cai[2,5]*

[1] School of Marine Sciences, Hainan University, Haikou 570228, China

[2] Department of Chemistry, Hong Kong Baptist University, Hong Kong, 999077, China

[3] Single-Cell Proteomics Research Center, ZJU-Hangzhou Global Scientific and Technological Innovation Center, Zhejiang University, Hangzhou 311215, China

[4] State Key Laboratory of Soil Pollution Control and Safety, Department of Environmental Science, Zhejiang University, Hangzhou 310058, China

[5] School of Chemistry, College of Science, Eastern Institute of Technology, Ningbo, Zhejiang 315100, China

*Corresponding Author

Prof. Jianing Wang

Email: justin.wang.4in1@gmail.com, Tel: 17301357252

Prof. Zongwei Cai

Email: zwcai@eitech.edu.cn, Tel: +852-34117070, Fax: +852-34117348

Table of Contents

Figure S1. Photographs of the ambient MSI platform based on the Knot-probe.................. 3
Figure S2. Photographs of fabrication processes of the Knot-probe. 4
Figure S3. Photographs of the liquid junction of different probes. 5
Figure S4. Photograph and schematic illustration of the Type 2 pneumatically-assisted Knot-probe. 6
Figure S5. Reproducibility of three Knot-probes fabricated by three operators for line scans. 7
Figure S6. Full total ion current (TIC) chromatogram and representative segments corresponding to Figure 2a. 8
Figure S7. Representative single-pixel mass spectra of lipids acquired in high-spatial-resolution imaging. 9
Figure S8. High-spatial-resolution imaging of small-molecule metabolites in mouse brain sections. 10
Figure S9. Tandem MSI for *sn*-positional isomers of PC 34:1 in a cerebellum section. 11
Figure S10. Mass spectra acquired from mouse brain sagittal sections using methanol-containing extraction solvents with different source dissociation voltage (SDV). 12
Figure S11. Native top-down MS/MS spectra of the 5+ precursor ion *m*/*z* 1691.1 ± 2........ 13
Figure S12. Native top-down MS/MS spectra of the 5+ precursor ion *m*/*z* 1713.9 ± 2. 14
Table S1. Comparison of the Knot-probe with nano-DESI probes. 15
Table S2. MSI Experimental parameters. 16
Table S3. Signal-dropout pixel fraction of selected ion images in this study. 17
Table S4. Putatively assigned lipids (*m*/*z* 500−1000, S/N > 10, error < 5 ppm) in mouse brain sections in negative ionization mode.. 18
Table S5. Putatively assigned lipids (*m*/*z* 500−1000, S/N > 10, error < 5 ppm) in mouse testis sections in negative ionization mode.. 22
Table S6. Putatively assigned small-molecule metabolites (*m*/*z* 100−500, S/N > 10, error < 5 ppm) in mouse brain sections in negative ionization mode. 25
References 29

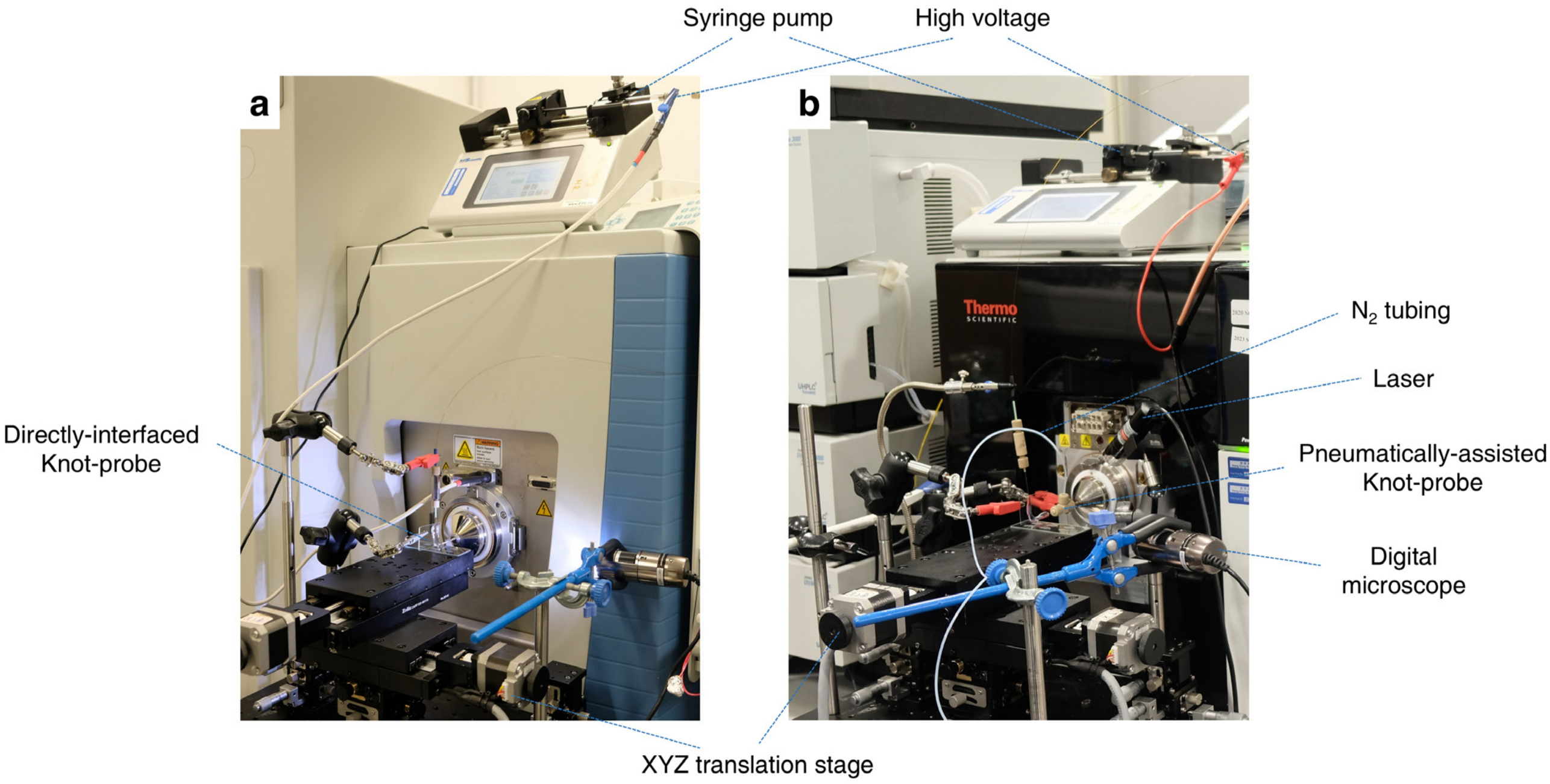

Figure S1. Photographs of the ambient MSI platform based on the Knot-probe. (a) Type 1 directly-interfaced Knot-probe. (b) Type 2 pneumatically-assisted Knot-probe.

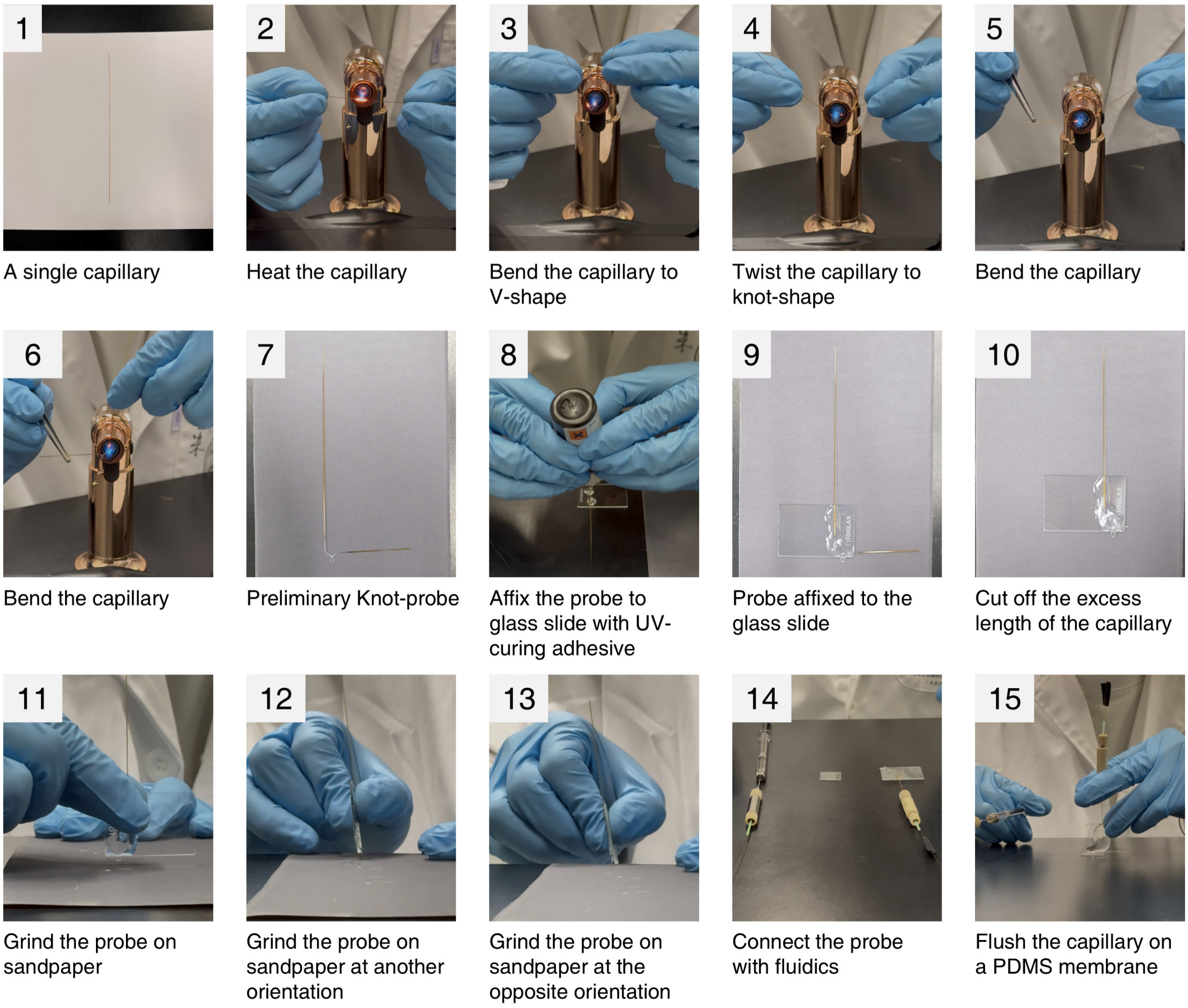

Figure S2. Photographs of fabrication processes of the Knot-probe.

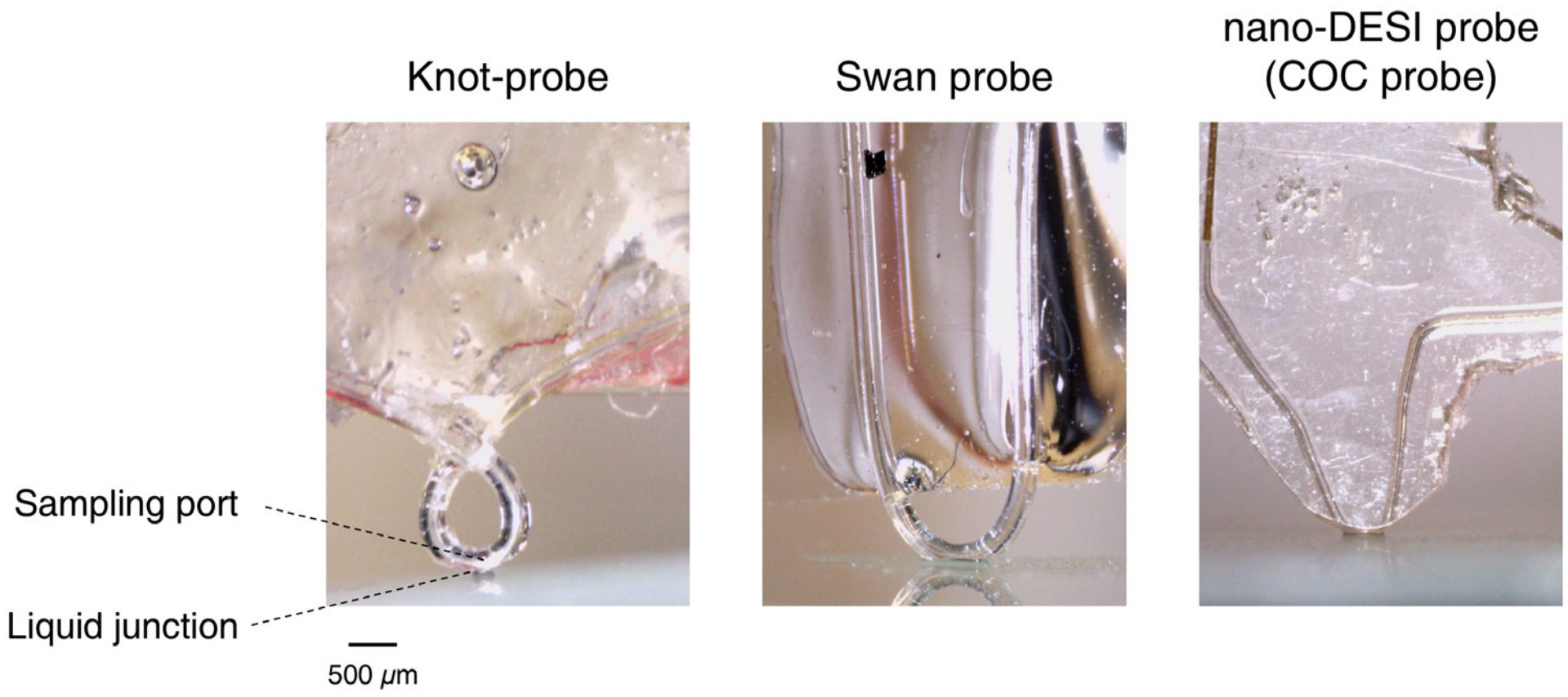

Figure S3. Photographs of the liquid junction of different probes. The Swan probe is fabricated according to Jin et al.[1] The nano-DESI COC probe is fabricated according to Li et al.[2]

a

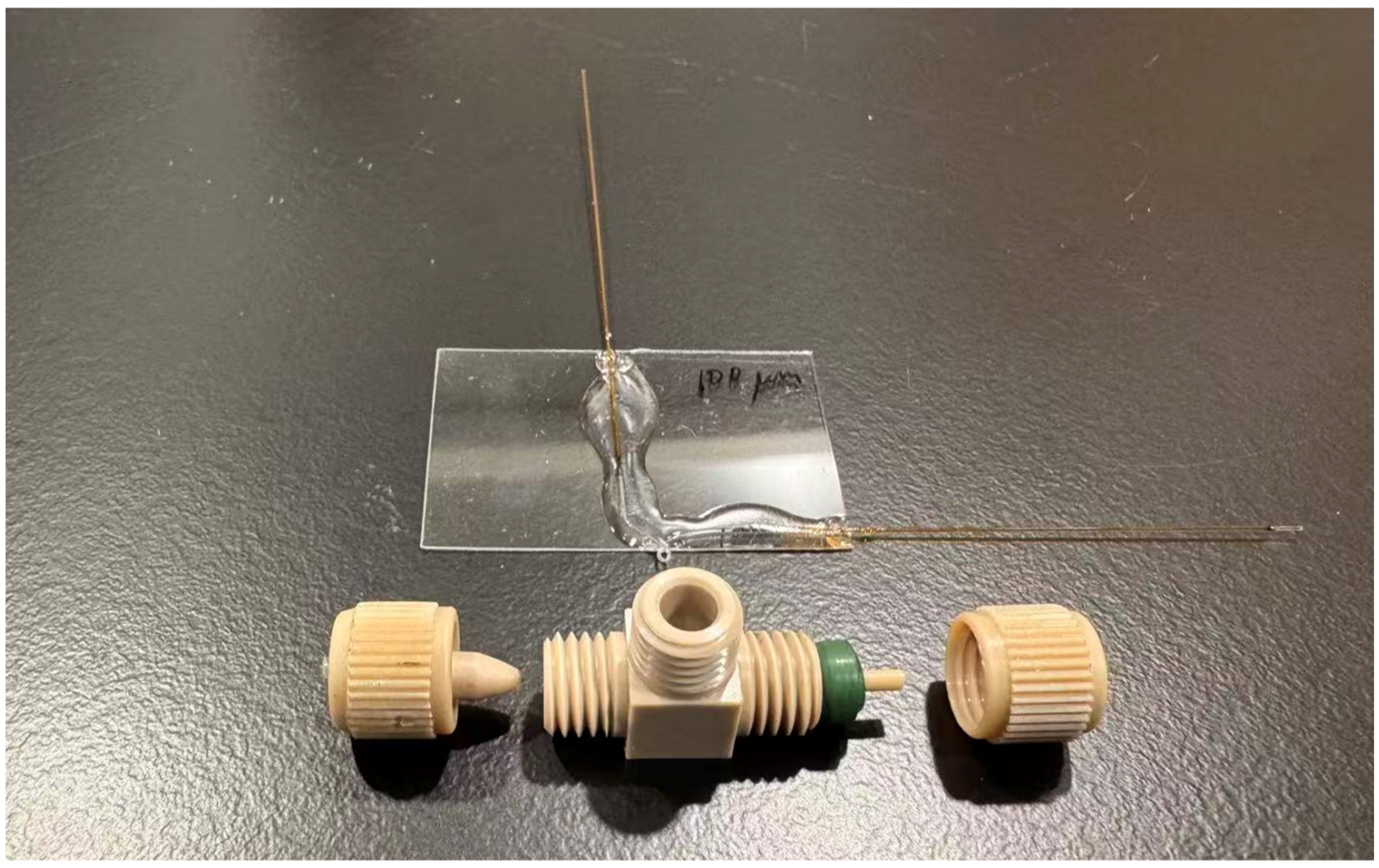

b

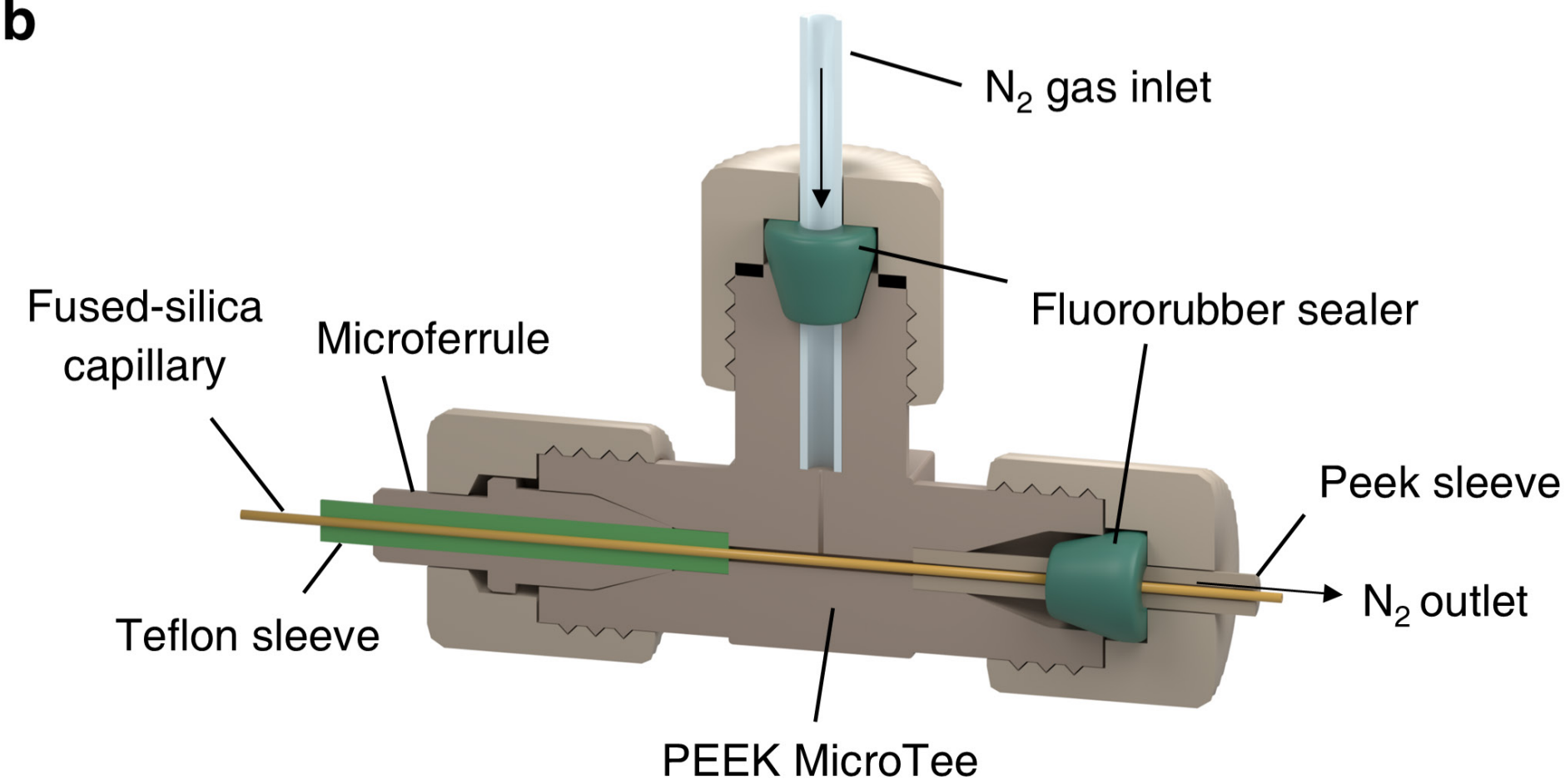

Figure S4. Photograph and schematic illustration of the Type 2 pneumatically-assisted Knot-probe.

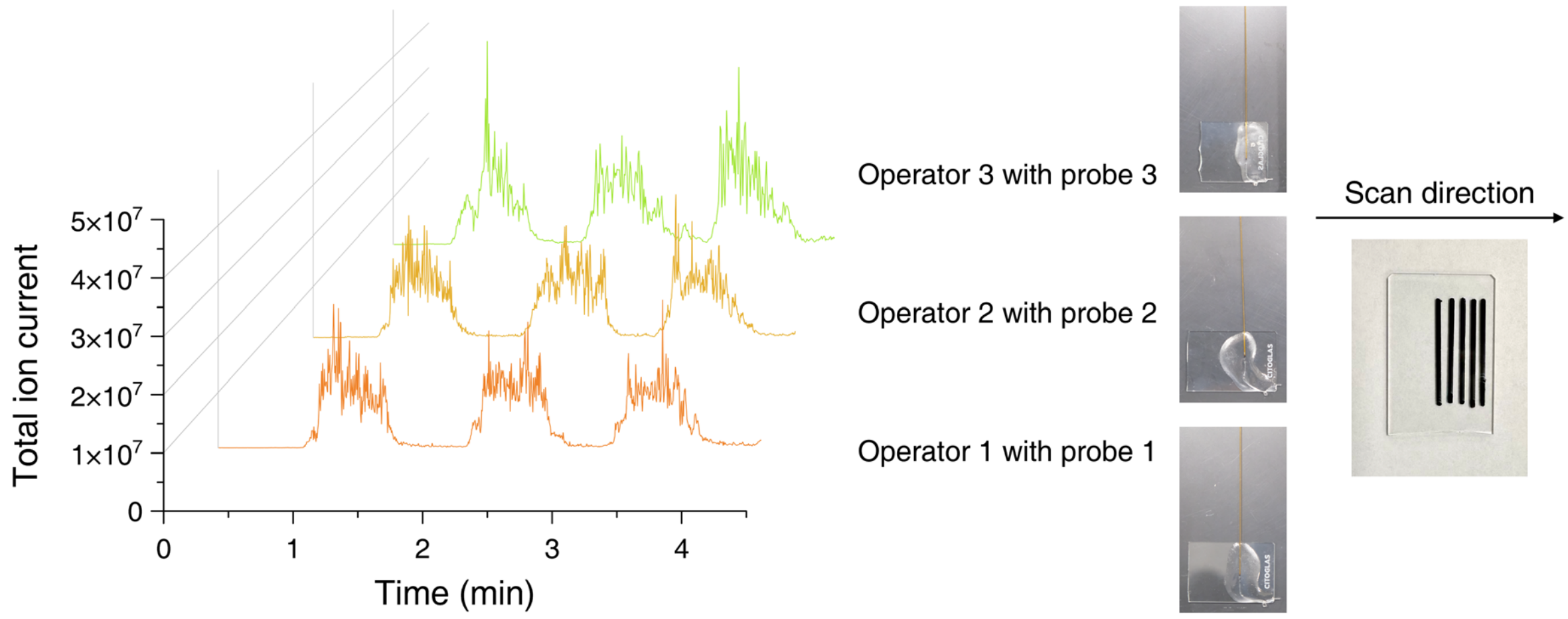

Figure S5. Reproducibility of three Knot-probes fabricated by three operators for line scans. The Knot-probes were scanned across the marker lines on the glass slide; scan speed, 60 μm s^{-1}.

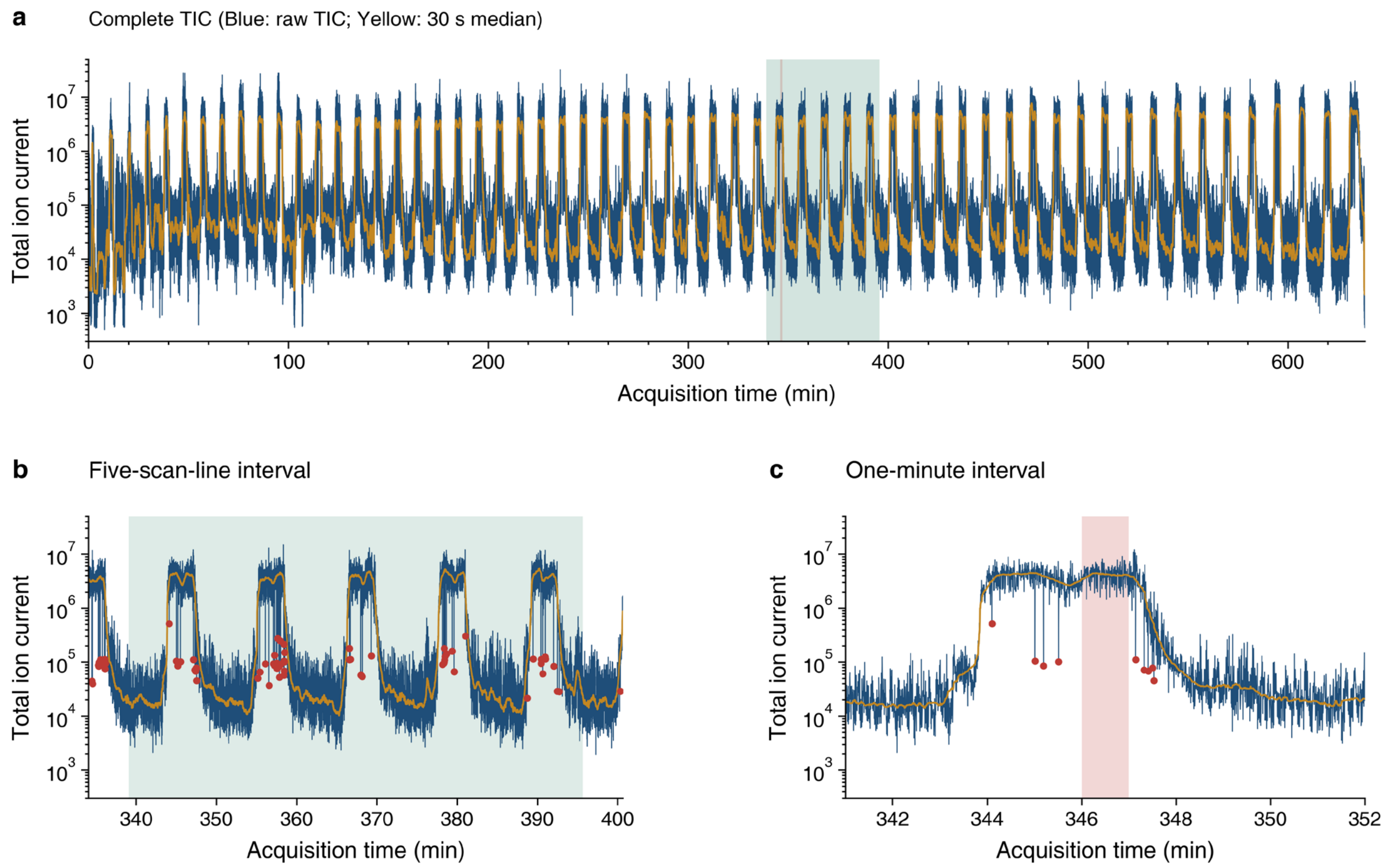

Figure S6. Full total ion current (TIC) chromatogram and representative segments corresponding to Figure 2a. (a) Full TIC chromatogram. (b) Five-scan-line interval. (c) One-minute interval.

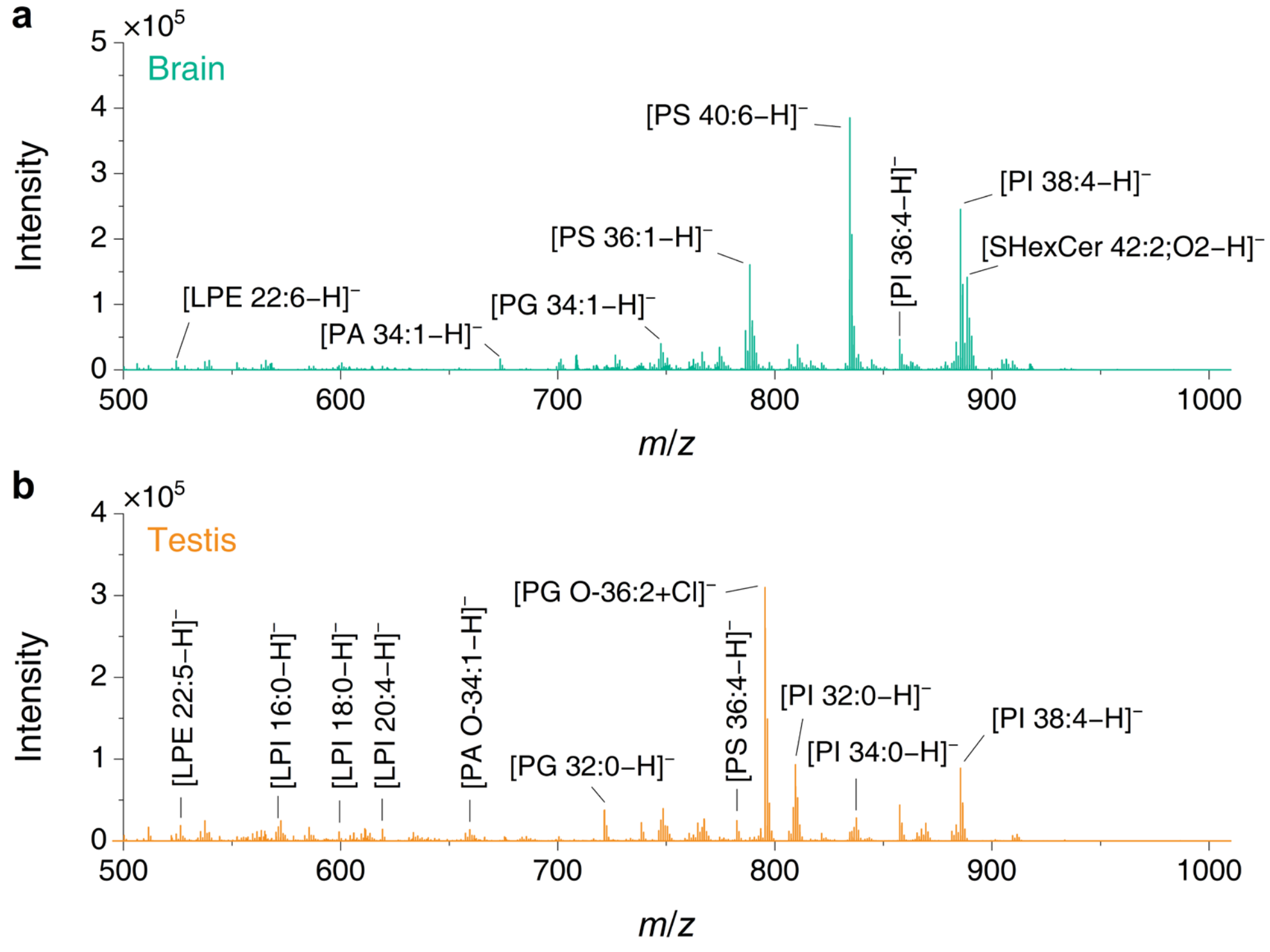

Figure S7. Representative single-pixel mass spectra of lipids acquired in high-spatial-resolution imaging. (a) Brain section, (b) testis section. Lipid assignments are putative.

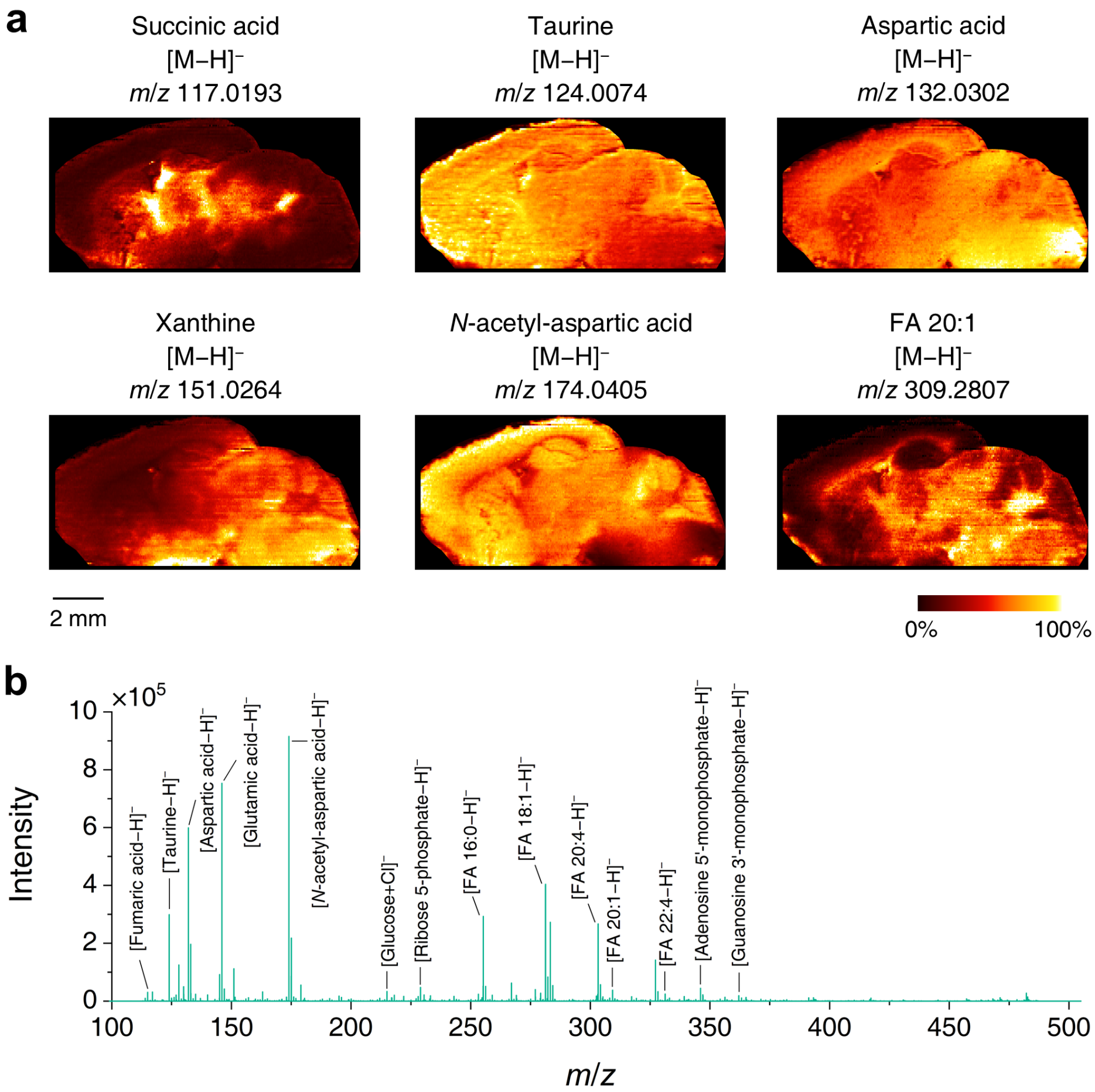

Figure S8. High-spatial-resolution imaging of small-molecule metabolites in mouse brain sections. Sample scan speed, 33 μm s^{-1}; vertical step size, 70 μm; in negative ionization mode. (a) Representative negative mode ion images of small-molecule metabolites in the mouse brain sagittal section. (b) Representative single-pixel mass spectrum showing small-molecule metabolites. Metabolite assignments are putative.

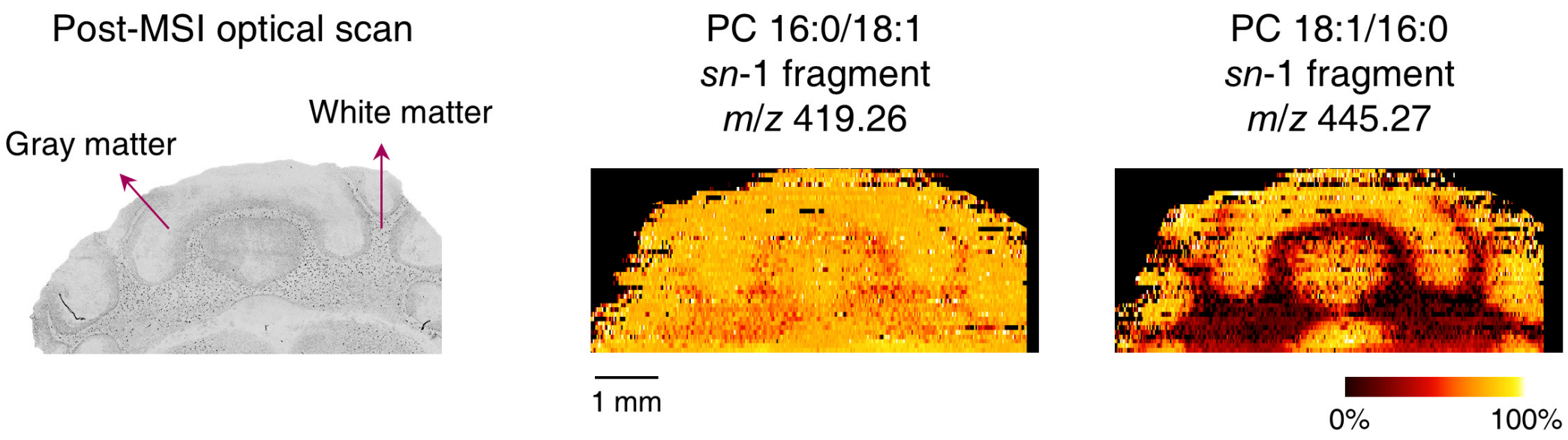

Figure S9. Tandem MSI for *sn*-positional isomers of PC 34:1 in a cerebellum section. Sample scan speed, 33 μm s^{-1}; vertical step size, 70 μm. Precursor *m*/*z* 820.5 ± 0.5, NCE 28%.

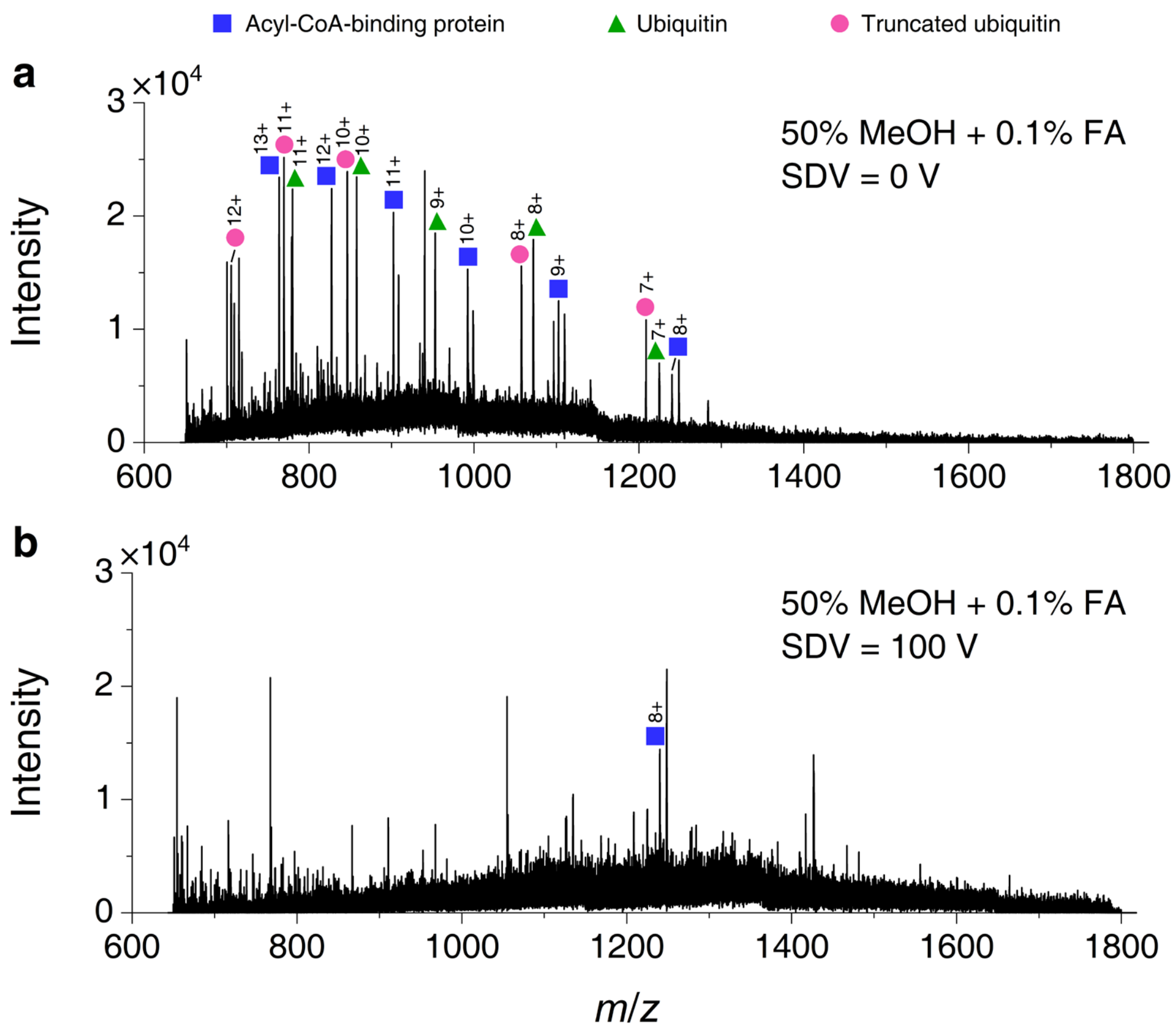

Figure S10. Mass spectra acquired from mouse brain sagittal sections using methanol-containing extraction solvents with different source dissociation voltage (SDV).

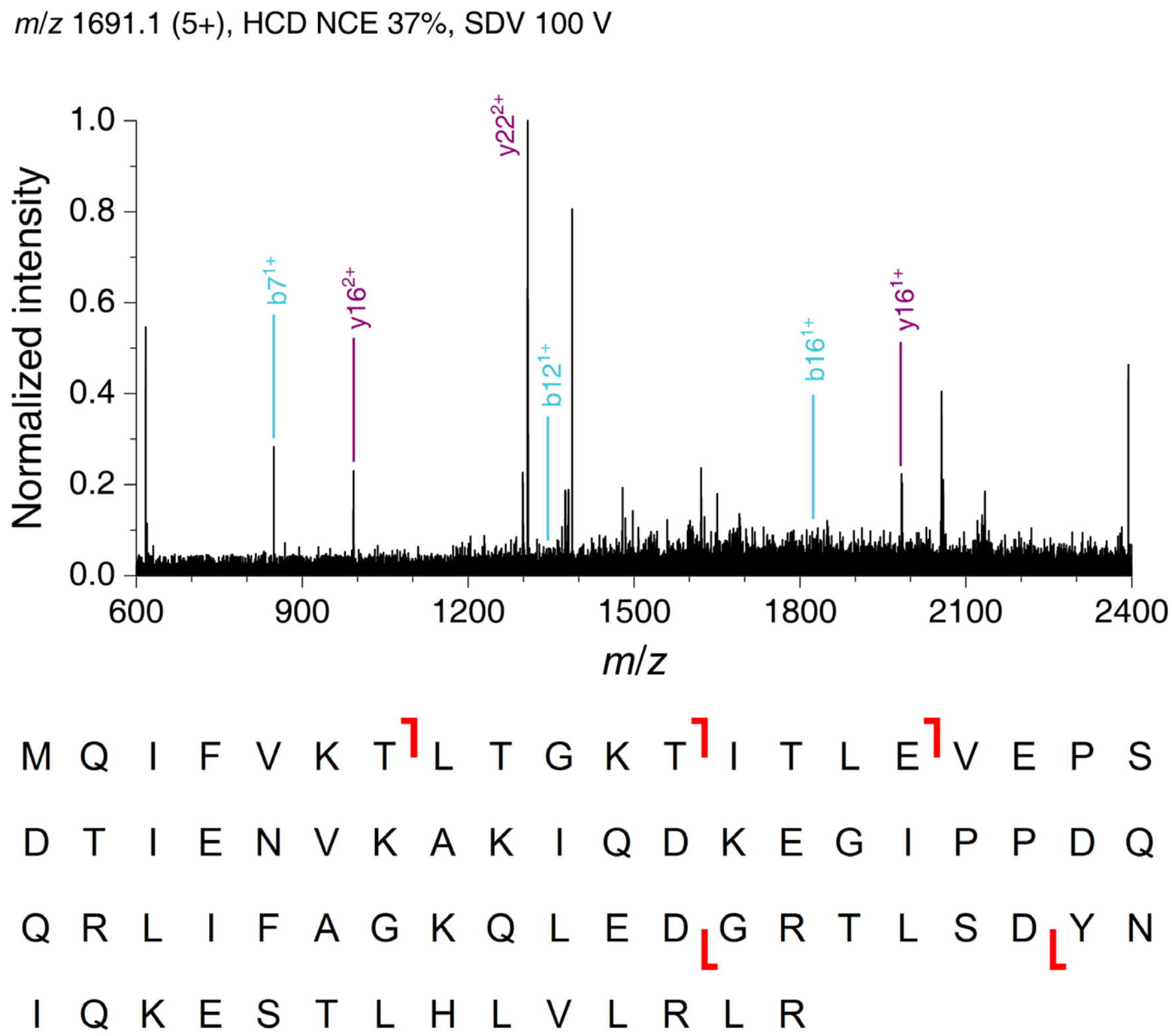

Figure S11. Native top-down MS/MS spectra of the 5+ precursor ion *m/z* 1691.1 ± 2.

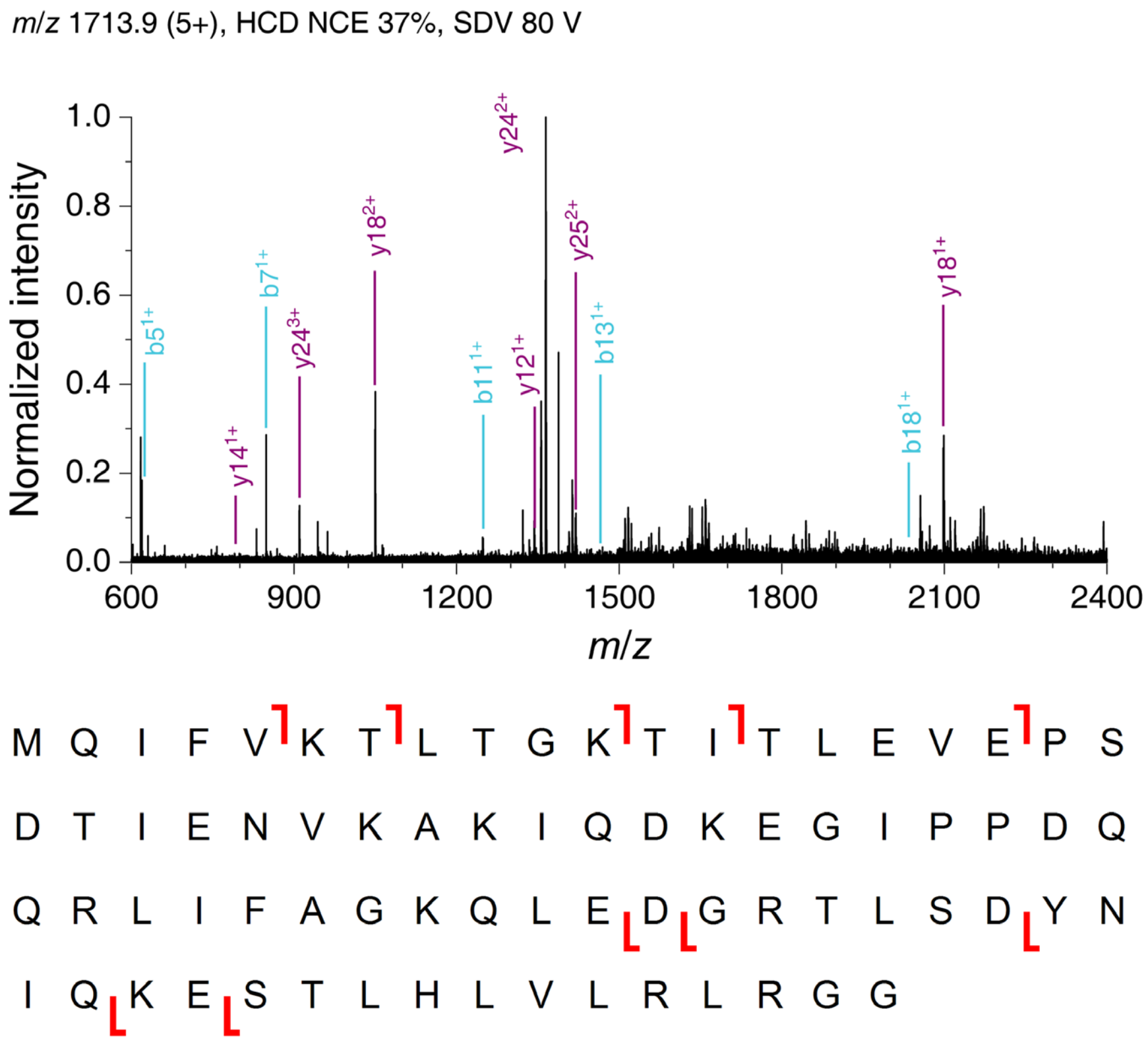

Figure S12. Native top-down MS/MS spectra of the 5+ precursor ion *m/z* 1713.9 ± 2.

Table S1. Comparison of the Knot-probe with nano-DESI probes.

Characteristics	Knot-probe (this study)	Two-capillary nano-DESI probe	Integrated microfluidic nano-DESI probe	Thermoplastic microfluidic nano-DESI probe
**Spatial resolution**	40 μm	100−150 μm,[3] 7−10 μm (finely pulled) [4,5]	8−10 μm,[6] 22−25 μm,[7] 25−30 μm,[8] ~20−30 μm[9]	5−7 μm,[2] 100−150 μm[10]
**Alignment degrees of freedom**	2	≥ 4	2	2
**Fabrication and setup time**	30 min	~4 h[5]	2−3 d,[7] ~1 d[9]	10 min (excluding 24 h cure time)[10]
**Scan speed**	26−160 μm s^{-1}	3−200 μm s^{-1}	20−400 μm s^{-1}	40 μm s^{-1}
**Main fabrication materials**	Fused-silica capillary	Fused-silica capillary	Glass or fused-silica microfluidic chip, or fused-silica capillary	Fused-silica capillary and cyclic olefin copolymer sheet
**Microfabrication requirements**	None	None	Photolithography or selective laser-assisted etching	Hot press machine[10] or furnace[2]
**Probe-to-probe reproducibility**	Good reproducibility reported for probes fabricated by three operators	Not reported	Not reported	Good reproducibility reported based on signal responses
**Maintenance**	Cleaning with a PDMS seal	Solvent flushing	Vacuum-assisted sequential rinsing[11]	Not reported
**Longest reported stable imaging run**	~11 h	~11 h[12]	16.3 h[6]	10−20 h[10]
**Longest reported probe lifetime**	Over one month	Over one week	Over several months[7]	Over several days[2]
**Applications**	Metabolites, lipids, lipid isomers, native proteins	Metabolites, lipids, lipid isomers, intact proteins, native proteins	Metabolites, lipids	Metabolites, lipids

Table S2. MSI Experimental parameters.

Experiments	Probe type	MS	Solvent and flow rate	Sample scan speed (μm s^{-1})	Vertical step size (μm)	Voltage and ion transfer tube temperature	S-lens RF	AGC and max. injection time	*m*/*z* range	Mass resolution at *m*/*z* 200	MS acquisition rate (s^{-1})
High-resolution MSI of lipids in brain	1	Q Exactive Focus	90% MeOH, 0.8 μL min^{-1}	37	70	−3.5 kV, 300 °C	60%	1×10^6, 100 ms	500−1000	70000	3.7
High-resolution MSI of lipids in testis	1	Q Exactive Focus	90% MeOH, 0.8 μL min^{-1}	37	70	−4 kV, 300 °C	60%	1×10^6, 100 ms	500−1000	70000	3.7
High-resolution MSI of metabolites in brain	1	Fusion Tribrid	90% MeOH, 1 μL min^{-1}	33	70	−4 kV, 250 °C	50%	3×10^6, 200 ms	100−500	60000	2.2
High-throughput MSI of lipids in brain*	1	Fusion Tribrid	90% MeOH, 2 μL min^{-1}	160	70	−4 kV, 300 °C	100%	3×10^6, 150 ms	500−1000	30000	4
High-resolution tandem MSI of lipid *sn*-positional isomers in brain	1	Fusion Tribrid	90% ACN + 5 mM NH_4HCO_3 and 0.5% NH_4OH, 0.8 μL min^{-1}	33 or 66	70	−4 kV, 300 °C	60%	1×10^6, 200 ms	400−900	15000	3.3 or 1.6
High-resolution native MSI of proteins in brain	2	Fusion Tribrid	200 mM NH_4OAc, 2.5 μL min^{-1} (50 psi nebulizing gas for solvents)	26	70	+1.7 kV, 250 °C	150%	5×10^6, 500 ms	1600−2200 or 1640−1720	60000	1.3

*Note that data in Figure 3c,d were acquired using the same instrument and parameters as "High-resolution MSI of lipids in brain" to facilitate comparison, except that the solvent flow rate was 2 μL min^{-1}.

Table S3. Signal-dropout pixel fraction of selected ion images in this study.

Ions	Figure	Signal-dropout pixel fraction
m/z 747.5180	Figure 2a	1.09%
m/z 786.5286	Figure 2a	0.31%
m/z 810.5286	Figure 2b	3.65%
m/z 834.5285	Figure 2b	0.61%
m/z 885.5490	Figure 2b	0.95%
m/z 760.5135	Figure 3b	2.50%
m/z 810.5286	Figure 3b	1.67%
m/z 865.5020	Figure 3b	2.80%
m/z 888.6230	Figure 3b	1.43%
m/z 419.26	Figure 4b	0.93%
m/z 445.27	Figure 4b	1.86%
m/z 1652.8	Figure 5c	3.86%
m/z 1691.1	Figure 5c	3.64%
m/z 1713.9	Figure 5c	0.48%

Table S4. Putatively assigned lipids (*m/z* 500−1000, S/N > 10, error < 5 ppm) in mouse brain sections in negative ionization mode.

Experimental *m/z*	Theoretical *m/z*	Error (ppm)	Putative assignment	Formula	Ion type*	Note**
500.2778	500.2783	−0.95	LPE 20:4	$C_{25}H_{44}NO_7P$	$[M-H]^-$	
506.3246	506.3252	−1.09	LPE 20:1	$C_{25}H_{50}NO_7P$	$[M-H]^-$	
509.2879	509.2885	−1.21	LPG 18:1	$C_{24}H_{47}O_9P$	$[M-H]^-$	
522.2829	522.2838	−1.66	LPS 18:1	$C_{24}H_{46}NO_9P$	$[M-H]^-$	
524.2778	524.2783	−0.94	LPE 22:6	$C_{27}H_{44}NO_7P$	$[M-H]^-$	
524.2986	524.2994	−1.44	LPS 18:0	$C_{24}H_{48}NO_9P$	$[M-H]^-$	Res.
528.3091	528.3096	−0.99	LPE 22:4	$C_{27}H_{48}NO_7P$	$[M-H]^-$	
531.2724	531.2729	−0.85	LPG 20:4	$C_{26}H_{45}O_9P$	$[M-H]^-$	
555.2722	555.2729	−1.32	LPG 22:6	$C_{28}H_{45}O_9P$	$[M-H]^-$	
568.2673	568.2681	−1.34	LPS 22:6	$C_{28}H_{44}NO_9P$	$[M-H]^-$	
571.2883	571.2889	−1.03	LPI 16:0	$C_{25}H_{49}O_{12}P$	$[M-H]^-$	
597.3037	597.3045	−1.26	LPI 18:1	$C_{27}H_{51}O_{12}P$	$[M-H]^-$	
598.4968	598.4972	−0.75	Cer 36:2;O2	$C_{36}H_{69}NO_3$	$[M+Cl]^-$	
599.3196	599.3202	−0.92	LPI 18:0	$C_{27}H_{53}O_{12}P$	$[M-H]^-$	
600.5131	600.5128	0.55	Cer 36:1;O2	$C_{36}H_{71}NO_3$	$[M+Cl]^-$	
614.2857	614.2866	−1.54	PE 22:2;O2	$C_{27}H_{50}NO_{10}P$	$[M+Cl]^-$	
619.2883	619.2889	−1.03	LPI 20:4	$C_{29}H_{49}O_{12}P$	$[M-H]^-$	
628.5434	628.5441	−1.14	Cer 38:1;O2	$C_{38}H_{75}NO_3$	$[M+Cl]^-$	
631.4728	631.4708	3.18	PA O-32:1	$C_{35}H_{69}O_7P$	$[M-H]^-$	Res.
647.4649	647.4657	−1.17	PA 32:0	$C_{35}H_{69}O_8P$	$[M-H]^-$	
657.4844	657.4865	−3.19	PA O-34:2	$C_{37}H_{71}O_7P$	$[M-H]^-$	
673.4810	673.4814	−0.66	PA 34:1	$C_{37}H_{71}O_8P$	$[M-H]^-$	
682.5913	682.5911	0.31	Cer 42:2;O2	$C_{42}H_{81}NO_3$	$[M+Cl]^-$	
683.5019	683.5021	−0.31	PA O-36:3	$C_{39}H_{73}O_7P$	$[M-H]^-$	
685.5176	685.5178	−0.29	PA O-36:2	$C_{39}H_{75}O_7P$	$[M-H]^-$	
687.4966	687.4970	−0.57	PA 35:1	$C_{38}H_{73}O_8P$	$[M-H]^-$	Res.
695.4657	695.4657	0.05	PA 36:4	$C_{39}H_{69}O_8P$	$[M-H]^-$	
698.5127	698.5130	−0.43	PE O-34:3	$C_{39}H_{74}NO_7P$	$[M-H]^-$	
699.4971	699.4970	0.09	PA 36:2	$C_{39}H_{73}O_8P$	$[M-H]^-$	
700.5279	700.5287	−1.18	PE O-34:2	$C_{39}H_{76}NO_7P$	$[M-H]^-$	
701.5108	701.5127	−2.75	PA 36:1	$C_{39}H_{75}O_8P$	$[M-H]^-$	
702.5434	702.5443	−1.27	PE O-34:1	$C_{39}H_{78}NO_7P$	$[M-H]^-$	Res.
711.5333	711.5334	−0.14	PA O-38:3	$C_{41}H_{77}O_7P$	$[M-H]^-$	
713.5479	713.5491	−1.68	PA O-38:2	$C_{41}H_{79}O_7P$	$[M-H]^-$	Res.
716.5232	716.5236	−0.50	PE 34:1	$C_{39}H_{76}NO_8P$	$[M-H]^-$	
718.5358	718.5392	−4.73	PE 34:0	$C_{39}H_{78}NO_8P$	$[M-H]^-$	
721.5027	721.5025	0.31	PG 32:0	$C_{38}H_{75}O_{10}P$	$[M-H]^-$	
722.5103	722.5130	−3.68	PE O-36:5	$C_{41}H_{74}NO_7P$	$[M-H]^-$	

Experimental *m*/*z*	Theoretical *m*/*z*	Error (ppm)	Putative assignment	Formula	Ion type*	Note**
723.4959	723.4970	−1.57	PA 38:4	$C_{41}H_{73}O_8P$	[M−H]⁻	Res.
724.5260	724.5287	−3.73	PE O-36:4	$C_{41}H_{76}NO_7P$	[M−H]⁻	
726.5437	726.5443	−0.76	PE O-36:3	$C_{41}H_{78}NO_7P$	[M−H]⁻	
728.5589	728.5600	−1.49	PE O-36:2	$C_{41}H_{80}NO_7P$	[M−H]⁻	
733.4821	733.4814	0.95	PA 39:6	$C_{42}H_{71}O_8P$	[M−H]⁻	Res.
738.5051	738.5079	−3.79	PE 36:4	$C_{41}H_{74}NO_8P$	[M−H]⁻	
742.5388	742.5392	−0.56	PE 36:2	$C_{41}H_{78}NO_8P$	[M−H]⁻	
744.5541	744.5549	−1.02	PE 36:1	$C_{41}H_{80}NO_8P$	[M−H]⁻	
745.5004	745.5025	−2.82	PG 34:2	$C_{40}H_{75}O_{10}P$	[M−H]⁻	
746.5128	746.5130	−0.24	PE O-38:7	$C_{43}H_{74}NO_7P$	[M−H]⁻	
747.5185	747.5182	0.37	PG 34:1	$C_{40}H_{77}O_{10}P$	[M−H]⁻	
748.5274	748.5287	−1.69	PE O-38:6	$C_{43}H_{76}NO_7P$	[M−H]⁻	Thru.
750.5432	750.5443	−1.43	PE O-38:5	$C_{43}H_{78}NO_7P$	[M−H]⁻	Thru.
754.5754	754.5756	−0.24	PE O-38:3	$C_{43}H_{82}NO_7P$	[M−H]⁻	
756.5896	756.5913	−2.29	PE O-38:2	$C_{43}H_{84}NO_7P$	[M−H]⁻	
760.5139	760.5134	0.71	PS 34:1	$C_{40}H_{76}NO_{10}P$	[M−H]⁻	
762.5060	762.5079	−2.43	PE 38:6	$C_{43}H_{74}NO_8P$	[M−H]⁻	
762.5650	762.5656	−0.84	HexCer 36:1;O2	$C_{42}H_{81}NO_8$	[M+Cl]⁻	Res.
764.5229	764.5236	−0.86	PE 38:5	$C_{43}H_{76}NO_8P$	[M−H]⁻	Res.
766.5388	766.5392	−0.50	PE 38:4	$C_{43}H_{78}NO_8P$	[M−H]⁻	
769.5027	769.5025	0.23	PG 36:4	$C_{42}H_{75}O_{10}P$	[M−H]⁻	Res.
770.5346	770.5342	0.52	PS O-36:3	$C_{42}H_{78}NO_9P$	[M−H]⁻	Res.
770.5699	770.5705	−0.75	PE 38:2	$C_{43}H_{82}NO_8P$	[M−H]⁻	
772.5283	772.5287	−0.46	PE O-40:8	$C_{45}H_{76}NO_7P$	[M−H]⁻	
773.4932	773.4894	4.88	PA 39:4	$C_{42}H_{75}O_8P$	[M+Cl]⁻	Res.
773.5326	773.5338	−1.57	PG 36:2	$C_{42}H_{79}O_{10}P$	[M−H]⁻	
774.5437	774.5443	−0.81	PE O-40:7	$C_{45}H_{78}NO_7P$	[M−H]⁻	
776.5565	776.5600	−4.54	PE O-40:6	$C_{45}H_{80}NO_7P$	[M−H]⁻	
778.5747	778.5756	−1.20	PE O-40:5	$C_{45}H_{82}NO_7P$	[M−H]⁻	
782.4984	782.4978	0.73	PS 36:4	$C_{42}H_{74}NO_{10}P$	[M−H]⁻	
784.5152	784.5134	2.29	PS 36:3	$C_{42}H_{76}NO_{10}P$	[M−H]⁻	
786.5290	786.5291	−0.07	PS 36:2	$C_{42}H_{78}NO_{10}P$	[M−H]⁻	
788.5453	788.5447	0.73	PS 36:1	$C_{42}H_{80}NO_{10}P$	[M−H]⁻	
790.5396	790.5392	0.48	PE 40:6	$C_{45}H_{78}NO_8P$	[M−H]⁻	
793.5057	793.5025	4.03	PG 38:6	$C_{44}H_{75}O_{10}P$	[M−H]⁻	
793.5540	793.5520	2.53	PA 40:1	$C_{43}H_{83}O_8P$	[M+Cl]⁻	Res.
795.5206	795.5182	2.97	PG 38:5	$C_{44}H_{77}O_{10}P$	[M−H]⁻	Res.
797.5330	797.5338	−1.06	PG 38:4	$C_{44}H_{79}O_{10}P$	[M−H]⁻	
799.5411	799.5414	−0.44	PA O-42:5	$C_{45}H_{81}O_7P$	[M+Cl]⁻	Res.
804.5353	804.5316	4.62	PE 38:3	$C_{43}H_{80}NO_8P$	[M+Cl]⁻	Res.

Experimental *m*/*z*	Theoretical *m*/*z*	Error (ppm)	Putative assignment	Formula	Ion type*	Note**
806.4985	806.4978	0.81	PS 38:6	$C_{44}H_{74}NO_{10}P$	$[M-H]^-$	Res.
806.5448	806.5458	−1.20	SHexCer 36:1;O2	$C_{42}H_{81}NO_{11}S$	$[M-H]^-$	
807.5022	807.5029	−0.91	PI 32:1	$C_{41}H_{77}O_{13}P$	$[M-H]^-$	Res.
808.5124	808.5134	−1.27	PS 38:5	$C_{44}H_{76}NO_{10}P$	$[M-H]^-$	
808.5503	808.5500	0.41	DGCC 36:5	$C_{46}H_{79}NO_8$	$[M+Cl]^-$	Res.
810.5292	810.5291	0.13	PS 38:4	$C_{44}H_{78}NO_{10}P$	$[M-H]^-$	
812.5418	812.5447	−3.55	PS 38:3	$C_{44}H_{80}NO_{10}P$	$[M-H]^-$	Res.
814.5613	814.5604	1.15	PS 38:2	$C_{44}H_{82}NO_{10}P$	$[M-H]^-$	
816.5780	816.5760	2.45	PS 38:1	$C_{44}H_{84}NO_{10}P$	$[M-H]^-$	
818.5331	818.5342	−1.39	PS O-40:7	$C_{46}H_{78}NO_9P$	$[M-H]^-$	Res.
818.5811	818.5836	−3.00	PE O-40:3	$C_{45}H_{86}NO_7P$	$[M+Cl]^-$	Res.
818.6265	818.6282	−2.08	HexCer 40:1;O2	$C_{46}H_{89}NO_8$	$[M+Cl]^-$	
821.5456	821.5469	−1.56	PG O-38:3	$C_{44}H_{83}O_9P$	$[M+Cl]^-$	
822.5793	822.5785	0.93	PC 36:1	$C_{44}H_{86}NO_8P$	$[M+Cl]^-$	Res.
832.5135	832.5134	0.09	PS 40:7	$C_{46}H_{76}NO_{10}P$	$[M-H]^-$	
832.6073	832.6075	−0.24	HexCer 40:2;O3	$C_{46}H_{87}NO_9$	$[M+Cl]^-$	
833.3869	833.3861	0.94	PI 28:2;O3	$C_{37}H_{67}O_{16}P$	$[M+Cl]^-$	
833.5172	833.5186	−1.68	PI 34:2	$C_{43}H_{79}O_{13}P$	$[M-H]^-$	
834.5286	834.5291	−0.58	PS 40:6	$C_{46}H_{78}NO_{10}P$	$[M-H]^-$	
835.5324	835.5342	−2.17	PI 34:1	$C_{43}H_{81}O_{13}P$	$[M-H]^-$	
838.5611	838.5604	0.80	PS 40:4	$C_{46}H_{82}NO_{10}P$	$[M-H]^-$	
841.5013	841.5025	−1.43	PG 42:10	$C_{48}H_{75}O_{10}P$	$[M-H]^-$	
842.5927	842.5917	1.17	PS 40:2	$C_{46}H_{86}NO_{10}P$	$[M-H]^-$	Res.
844.6058	844.6073	−1.72	PS 40:1	$C_{46}H_{88}NO_{10}P$	$[M-H]^-$	Res.
844.6439	844.6439	0.04	HexCer 42:2;O2	$C_{48}H_{91}NO_8$	$[M+Cl]^-$	
846.5648	846.5655	−0.83	PS O-42:7	$C_{48}H_{82}NO_9P$	$[M-H]^-$	
848.6414	848.6388	3.06	HexCer 41:1;O3	$C_{47}H_{91}NO_9$	$[M+Cl]^-$	
848.8066	848.8077	−1.33	Cer 54:1;O4	$C_{54}H_{107}NO_5$	$[M-H]^-$	
855.5008	855.5029	−2.45	PI 36:5	$C_{45}H_{77}O_{13}P$	$[M-H]^-$	
857.5178	857.5186	−0.99	PI 36:4	$C_{45}H_{79}O_{13}P$	$[M-H]^-$	
858.5207	858.5174	3.85	SHexCer 36:1;O3	$C_{42}H_{81}NO_{12}S$	$[M+Cl]^-$	
858.6213	858.6231	−2.10	HexCer 42:3;O3	$C_{48}H_{89}NO_9$	$[M+Cl]^-$	
860.5920	860.5942	−2.58	PE 42:3	$C_{47}H_{88}NO_8P$	$[M+Cl]^-$	Res.
860.6378	860.6388	−1.21	HexCer 42:2;O3	$C_{48}H_{91}NO_9$	$[M+Cl]^-$	Res.
861.5488	861.5499	−1.24	PI 36:2	$C_{45}H_{83}O_{13}P$	$[M-H]^-$	
862.6071	862.6084	−1.46	SHexCer 40:1;O2	$C_{46}H_{89}NO_{11}S$	$[M-H]^-$	Res.
862.6581	862.6544	4.24	HexCer 42:1;O3	$C_{48}H_{93}NO_9$	$[M+Cl]^-$	Res.
863.5644	863.5655	−1.25	PI 36:1	$C_{45}H_{85}O_{13}P$	$[M-H]^-$	Res.
865.5030	865.5025	0.58	PG 44:12	$C_{50}H_{75}O_{10}P$	$[M-H]^-$	
865.5737	865.5731	0.68	PG 40:2	$C_{46}H_{87}O_{10}P$	$[M+Cl]^-$	Res.

Experimental *m*/*z*	Theoretical *m*/*z*	Error (ppm)	Putative assignment	Formula	Ion type*	Note**
870.6229	870.6230	−0.16	PS 42:2	$C_{48}H_{90}NO_{10}P$	$[M-H]^-$	Res.
871.5329	871.5342	−1.50	PI 37:4	$C_{46}H_{81}O_{13}P$	$[M-H]^-$	Res.
872.6369	872.6386	−1.93	PS 42:1	$C_{48}H_{92}NO_{10}P$	$[M-H]^-$	
874.6084	874.6098	−1.56	PC 40:3	$C_{48}H_{90}NO_{8}P$	$[M+Cl]^-$	
876.6244	876.6255	−1.28	PC 40:2	$C_{48}H_{92}NO_{8}P$	$[M+Cl]^-$	
878.4979	878.4978	0.06	PS 44:12	$C_{50}H_{74}NO_{10}P$	$[M-H]^-$	
878.6011	878.6033	−2.50	SHexCer 40:1;O3	$C_{46}H_{89}NO_{12}S$	$[M-H]^-$	
879.5009	879.5029	−2.25	PI 38:7	$C_{47}H_{77}O_{13}P$	$[M-H]^-$	
881.5186	881.5186	−0.01	PI 38:6	$C_{47}H_{79}O_{13}P$	$[M-H]^-$	
882.5261	882.5291	−3.39	PS 44:10	$C_{50}H_{78}NO_{10}P$	$[M-H]^-$	Thru.
883.5338	883.5342	−0.47	PI 38:5	$C_{47}H_{81}O_{13}P$	$[M-H]^-$	
885.5492	885.5499	−0.80	PI 38:4	$C_{47}H_{83}O_{13}P$	$[M-H]^-$	
886.6059	886.6098	−4.40	PE 44:4	$C_{49}H_{90}NO_{8}P$	$[M+Cl]^-$	Res.
887.5566	887.5574	−0.87	PG 42:5	$C_{48}H_{85}O_{10}P$	$[M+Cl]^-$	Res.
888.6239	888.6240	−0.16	SHexCer 42:2;O2	$C_{48}H_{91}NO_{11}S$	$[M-H]^-$	
890.6353	890.6397	−4.94	SHexCer 42:1;O2	$C_{48}H_{93}NO_{11}S$	$[M-H]^-$	
902.6385	902.6411	−2.84	PC 42:3	$C_{50}H_{94}NO_{8}P$	$[M+Cl]^-$	Res.
904.6173	904.6189	−1.75	SHexCer 42:2;O3	$C_{48}H_{91}NO_{12}S$	$[M-H]^-$	
906.6329	906.6346	−1.87	SHexCer 42:1;O3	$C_{48}H_{93}NO_{12}S$	$[M-H]^-$	
907.5331	907.5342	−1.21	PI 40:7	$C_{49}H_{81}O_{13}P$	$[M-H]^-$	
909.5491	909.5499	−0.90	PI 40:6	$C_{49}H_{83}O_{13}P$	$[M-H]^-$	
911.5604	911.5574	3.25	PG 44:7	$C_{50}H_{85}O_{10}P$	$[M+Cl]^-$	
913.5795	913.5812	−1.82	PI 40:4	$C_{49}H_{87}O_{13}P$	$[M-H]^-$	

* $[M+Cl]^-$ ions were manually validated by the presence and abundance ratio of their ^{37}Cl isotopologue peaks.

** Lipids only assigned in high-resolution or high-throughput imaging experiments are denoted by Res. or Thru., respectively.

Table S5. Putatively assigned lipids (*m/z* 500−1000, S/N > 10, error < 5 ppm) in mouse testis sections in negative ionization mode.

Experimental *m/z*	Theoretical *m/z*	Error (ppm)	Putative assignment	Formula	Ion type*
500.2775	500.2783	−1.68	LPE 20:4	$C_{25}H_{44}NO_7P$	$[M-H]^-$
509.2872	509.2885	−2.65	LPG 18:1	$C_{24}H_{47}O_9P$	$[M-H]^-$
509.4563	509.4575	−2.38	FA 32:1;O2	$C_{32}H_{62}O_4$	$[M-H]^-$
522.2828	522.2838	−1.92	LPS 18:1	$C_{24}H_{46}NO_9P$	$[M-H]^-$
524.2772	524.2783	−2.18	LPE 22:6	$C_{27}H_{44}NO_7P$	$[M-H]^-$
524.2969	524.2994	−4.68	LPS 18:0	$C_{24}H_{48}NO_9P$	$[M-H]^-$
526.2929	526.2939	−1.99	LPE 22:5	$C_{27}H_{46}NO_7P$	$[M-H]^-$
528.3080	528.3096	−2.98	LPE 22:4	$C_{27}H_{48}NO_7P$	$[M-H]^-$
530.3008	530.3019	−2.12	LPC 16:0	$C_{24}H_{50}NO_7P$	$[M+Cl]^-$
536.5038	536.5048	−1.88	Cer 34:1;O2	$C_{34}H_{67}NO_3$	$[M-H]^-$
537.4881	537.4888	−1.35	FAHFA 34:0;O	$C_{34}H_{66}O_4$	$[M-H]^-$
544.2671	544.2681	−1.86	LPS 20:4	$C_{26}H_{44}NO_9P$	$[M-H]^-$
554.3237	554.3254	−3.11	DGTS 20:5	$C_{30}H_{49}NO_6$	$[M+Cl]^-$
555.2718	555.2729	−2.00	LPG 22:6	$C_{28}H_{45}O_9P$	$[M-H]^-$
556.3171	556.3175	−0.77	LPC 18:1	$C_{26}H_{52}NO_7P$	$[M+Cl]^-$
565.5194	565.5201	−1.22	FAHFA 36:0;O	$C_{36}H_{70}O_4$	$[M-H]^-$
568.2671	568.2681	−1.79	LPS 22:6	$C_{28}H_{44}NO_9P$	$[M-H]^-$
569.2708	569.2732	−4.23	LPI 16:1	$C_{25}H_{47}O_{12}P$	$[M-H]^-$
571.2874	571.2889	−2.70	LPI 16:0	$C_{25}H_{49}O_{12}P$	$[M-H]^-$
578.3010	578.3019	−1.61	LPC 20:4	$C_{28}H_{50}NO_7P$	$[M+Cl]^-$
580.3627	580.3620	1.25	LPS 22:0	$C_{28}H_{56}NO_9P$	$[M-H]^-$
587.5036	587.5045	−1.48	FAHFA 38:3;O	$C_{38}H_{68}O_4$	$[M-H]^-$
597.3034	597.3045	−1.80	LPI 18:1	$C_{27}H_{51}O_{12}P$	$[M-H]^-$
599.3194	599.3202	−1.25	LPI 18:0	$C_{27}H_{53}O_{12}P$	$[M-H]^-$
602.3011	602.3019	−1.37	LPC 22:6	$C_{30}H_{50}NO_7P$	$[M+Cl]^-$
604.3173	604.3175	−0.27	LPC 22:5	$C_{30}H_{52}NO_7P$	$[M+Cl]^-$
619.2880	619.2889	−1.38	LPI 20:4	$C_{29}H_{49}O_{12}P$	$[M-H]^-$
631.4721	631.4708	2.02	PA O-32:1	$C_{35}H_{69}O_7P$	$[M-H]^-$
633.4876	633.4865	1.68	PA O-32:0	$C_{35}H_{71}O_7P$	$[M-H]^-$
643.2880	643.2889	−1.38	LPI 22:6	$C_{31}H_{49}O_{12}P$	$[M-H]^-$
655.4719	655.4708	1.69	PA O-34:3	$C_{37}H_{69}O_7P$	$[M-H]^-$
657.4873	657.4865	1.19	PA O-34:2	$C_{37}H_{71}O_7P$	$[M-H]^-$
659.5025	659.5021	0.58	PA O-34:1	$C_{37}H_{73}O_7P$	$[M-H]^-$
661.5182	661.5178	0.58	PA O-34:0	$C_{37}H_{75}O_7P$	$[M-H]^-$
666.3169	666.3179	−1.43	PE 26:4;O2	$C_{31}H_{54}NO_{10}P$	$[M+Cl]^-$
682.5903	682.5911	−1.22	Cer 42:2;O2	$C_{42}H_{81}NO_3$	$[M+Cl]^-$
687.5329	687.5334	−0.72	PA O-36:1	$C_{39}H_{77}O_7P$	$[M-H]^-$
689.5495	689.5491	0.64	PA O-36:0	$C_{39}H_{79}O_7P$	$[M-H]^-$

Experimental *m*/*z*	Theoretical *m*/*z*	Error (ppm)	Putative assignment	Formula	Ion type*
700.5279	700.5287	−1.15	PE O-34:2	$C_{39}H_{76}NO_7P$	$[M-H]^-$
707.5242	707.5233	1.24	PG O-32:0	$C_{38}H_{77}O_9P$	$[M-H]^-$
716.5227	716.5236	−1.26	PE 34:1	$C_{39}H_{76}NO_8P$	$[M-H]^-$
721.5020	721.5025	−0.73	PG 32:0	$C_{38}H_{75}O_{10}P$	$[M-H]^-$
731.5247	731.5233	1.90	PG O-34:2	$C_{40}H_{77}O_9P$	$[M-H]^-$
733.5404	733.5389	2.10	PG O-34:1	$C_{40}H_{79}O_9P$	$[M-H]^-$
737.5360	737.5370	−1.35	SM 34:1;O2	$C_{39}H_{79}N_2O_6P$	$[M+Cl]^-$
738.5073	738.5079	−0.84	PE 36:4	$C_{41}H_{74}NO_8P$	$[M-H]^-$
743.5972	743.5960	1.66	PA O-40:1	$C_{43}H_{85}O_7P$	$[M-H]^-$
746.5123	746.5130	−0.95	PE O-38:7	$C_{43}H_{74}NO_7P$	$[M-H]^-$
747.5172	747.5182	−1.37	PG 34:1	$C_{40}H_{77}O_{10}P$	$[M-H]^-$
748.5268	748.5287	−2.57	PE O-38:6	$C_{43}H_{76}NO_7P$	$[M-H]^-$
750.5427	750.5443	−2.09	PE O-38:5	$C_{43}H_{78}NO_7P$	$[M-H]^-$
759.6252	759.6273	−2.77	PA O-41:0	$C_{44}H_{89}O_7P$	$[M-H]^-$
760.5126	760.5134	−1.09	PS 34:1	$C_{40}H_{76}NO_{10}P$	$[M-H]^-$
762.5069	762.5079	−1.36	PE 38:6	$C_{43}H_{74}NO_8P$	$[M-H]^-$
764.5224	764.5236	−1.54	PE 38:5	$C_{43}H_{76}NO_8P$	$[M-H]^-$
766.5019	766.5029	−1.26	PS O-36:5	$C_{42}H_{74}NO_9P$	$[M-H]^-$
766.5377	766.5392	−2.02	PE 38:4	$C_{43}H_{78}NO_8P$	$[M-H]^-$
767.4981	767.4999	−2.38	PG O-34:2	$C_{40}H_{77}O_9P$	$[M+Cl]^-$
769.5022	769.5025	−0.42	PG 36:4	$C_{42}H_{75}O_{10}P$	$[M-H]^-$
774.5417	774.5443	−3.38	PE O-40:7	$C_{45}H_{78}NO_7P$	$[M-H]^-$
776.5224	776.5236	−1.61	PE 39:6	$C_{44}H_{76}NO_8P$	$[M-H]^-$
776.5580	776.5600	−2.59	PE O-40:6	$C_{45}H_{80}NO_7P$	$[M-H]^-$
778.5385	778.5392	−0.94	PE 39:5	$C_{44}H_{78}NO_8P$	$[M-H]^-$
778.5736	778.5756	−2.58	PE O-40:5	$C_{45}H_{82}NO_7P$	$[M-H]^-$
782.4969	782.4978	−1.18	PS 36:4	$C_{42}H_{74}NO_{10}P$	$[M-H]^-$
786.5281	786.5291	−1.23	PS 36:2	$C_{42}H_{78}NO_{10}P$	$[M-H]^-$
788.5453	788.5447	0.76	PS 36:1	$C_{42}H_{80}NO_{10}P$	$[M-H]^-$
790.5382	790.5392	−1.28	PE 40:6	$C_{45}H_{78}NO_8P$	$[M-H]^-$
792.5177	792.5185	−0.95	PS O-38:6	$C_{44}H_{76}NO_9P$	$[M-H]^-$
792.5533	792.5549	−2.03	PE 40:5	$C_{45}H_{80}NO_8P$	$[M-H]^-$
793.5143	793.5156	−1.66	PG O-36:3	$C_{42}H_{79}O_9P$	$[M+Cl]^-$
795.5291	795.5312	−2.64	PG O-36:2	$C_{42}H_{81}O_9P$	$[M+Cl]^-$
797.5342	797.5338	0.49	PG 38:4	$C_{44}H_{79}O_{10}P$	$[M-H]^-$
806.4972	806.4978	−0.71	PS 38:6	$C_{44}H_{74}NO_{10}P$	$[M-H]^-$
807.5001	807.5029	−3.52	PI 32:1	$C_{41}H_{77}O_{13}P$	$[M-H]^-$
808.5124	808.5134	−1.28	PS 38:5	$C_{44}H_{76}NO_{10}P$	$[M-H]^-$
809.5169	809.5186	−2.14	PI 32:0	$C_{41}H_{79}O_{13}P$	$[M-H]^-$
816.5304	816.5316	−1.44	PE 39:4	$C_{44}H_{80}NO_8P$	$[M+Cl]^-$

Experimental *m*/*z*	Theoretical *m*/*z*	Error (ppm)	Putative assignment	Formula	Ion type*
818.5330	818.5342	−1.43	PS O-40:7	$C_{46}H_{78}NO_9P$	[M−H]⁻
821.5447	821.5469	−2.74	PG O-38:3	$C_{44}H_{83}O_9P$	[M+Cl]⁻
823.5601	823.5625	−2.90	PG O-38:2	$C_{44}H_{85}O_9P$	[M+Cl]⁻
833.5173	833.5186	−1.59	PI 34:2	$C_{43}H_{79}O_{13}P$	[M−H]⁻
834.5282	834.5291	−1.10	PS 40:6	$C_{46}H_{78}NO_{10}P$	[M−H]⁻
835.5322	835.5342	−2.42	PI 34:1	$C_{43}H_{81}O_{13}P$	[M−H]⁻
836.5425	836.5447	−2.62	PS 40:5	$C_{46}H_{80}NO_{10}P$	[M−H]⁻
837.5485	837.5499	−1.70	PI 34:0	$C_{43}H_{83}O_{13}P$	[M−H]⁻
841.5019	841.5025	−0.74	PG 42:10	$C_{48}H_{75}O_{10}P$	[M−H]⁻
843.5411	843.5393	2.15	PI O-36:4	$C_{45}H_{81}O_{12}P$	[M−H]⁻
857.5177	857.5186	−1.02	PI 36:4	$C_{45}H_{79}O_{13}P$	[M−H]⁻
864.5735	864.5760	−2.95	PS 42:5	$C_{48}H_{84}NO_{10}P$	[M−H]⁻
865.5013	865.5025	−1.44	PG 44:12	$C_{50}H_{75}O_{10}P$	[M−H]⁻
869.5310	869.5338	−3.22	PG 44:10	$C_{50}H_{79}O_{10}P$	[M−H]⁻
871.5379	871.5342	4.25	PI 37:4	$C_{46}H_{81}O_{13}P$	[M−H]⁻
881.5177	881.5186	−1.06	PI 38:6	$C_{47}H_{79}O_{13}P$	[M−H]⁻
883.5333	883.5342	−1.04	PI 38:5	$C_{47}H_{81}O_{13}P$	[M−H]⁻
885.5490	885.5499	−1.00	PI 38:4	$C_{47}H_{83}O_{13}P$	[M−H]⁻
909.5488	909.5499	−1.22	PI 40:6	$C_{49}H_{83}O_{13}P$	[M−H]⁻
911.5635	911.5655	−2.24	PI 40:5	$C_{49}H_{85}O_{13}P$	[M−H]⁻

* [M+Cl]⁻ ions were manually validated by the presence and abundance ratio of their ^{37}Cl isotopologue peaks.

Table S6. Putatively assigned small-molecule metabolites (*m/z* 100−500, S/N > 10, error < 5 ppm) in mouse brain sections in negative ionization mode.

Experimental *m/z*	Theoretical *m/z*	Error (ppm)	Putative assignment	Formula	Ion type*
111.0198	111.0200	−1.80	Uracil	$C_4H_4N_2O_2$	$[M-H]^-$
115.0036	115.0037	−0.87	Fumaric acid	$C_4H_4O_4$	$[M-H]^-$
115.0396	115.0401	−4.51	3-Oxopentanoic acid	$C_5H_8O_3$	$[M-H]^-$
116.0713	116.0717	−3.46	Valine	$C_5H_{11}NO_2$	$[M-H]^-$
117.0193	117.0193	0.00	Succinic acid	$C_4H_6O_4$	$[M-H]^-$
117.0553	117.0557	−3.57	3-Hydroxyisovaleric acid	$C_5H_{10}O_3$	$[M-H]^-$
118.0506	118.0510	−3.11	Threonine	$C_4H_9NO_3$	$[M-H]^-$
119.0345	119.0350	−4.05	Erythrulose	$C_4H_8O_4$	$[M-H]^-$
120.0124	120.0125	−0.83	Cysteine	$C_3H_7NO_2S$	$[M-H]^-$
121.0291	121.0295	−3.33	4-Hydroxybenzaldehyde	$C_7H_6O_2$	$[M-H]^-$
122.9852	122.9853	−0.81	Phosphonoacetaldehyde	$C_2H_5O_4P$	$[M-H]^-$
124.0074	124.0074	0.00	Taurine	$C_2H_7NO_3S$	$[M-H]^-$
127.0509	127.0513	−3.48	Dihydrothymine	$C_5H_8N_2O_2$	$[M-H]^-$
128.0349	128.0353	−3.26	Pyroglutamic acid	$C_5H_7NO_3$	$[M-H]^-$
129.0190	129.0193	−2.58	2,5-Dioxopentanoate	$C_5H_6O_4$	$[M-H]^-$
129.0552	129.0557	−4.01	Ketoleucine	$C_6H_{10}O_3$	$[M-H]^-$
129.0917	129.0921	−3.12	Heptanoic acid	$C_7H_{14}O_2$	$[M-H]^-$
130.0506	130.0510	−2.82	*N*-Acetyl-alanine	$C_5H_9NO_3$	$[M-H]^-$
130.0622	130.0622	0.00	Creatine	$C_4H_9N_3O_2$	$[M-H]^-$
130.0873	130.0874	−0.77	Leucine	$C_6H_{13}NO_2$	$[M-H]^-$
131.0350	131.0350	0.00	Glutaric acid	$C_5H_8O_4$	$[M-H]^-$
131.0458	131.0462	−3.17	Asparagine	$C_4H_8N_2O_3$	$[M-H]^-$
132.0302	132.0302	0.00	Aspartic acid	$C_4H_7NO_4$	$[M-H]^-$
133.0142	133.0142	0.00	Malic acid	$C_4H_6O_5$	$[M-H]^-$
133.0501	133.0506	−4.00	2-Deoxypentose	$C_5H_{10}O_4$	$[M-H]^-$
134.0471	134.0472	−0.75	Adenine	$C_5H_5N_5$	$[M-H]^-$
135.0300	135.0299	0.76	Threonic acid	$C_4H_8O_5$	$[M-H]^-$
135.0311	135.0312	−0.74	Hypoxanthine	$C_5H_4N_4O$	$[M-H]^-$
138.0192	138.0197	−3.38	Hydroxynicotinic acid	$C_6H_5NO_3$	$[M-H]^-$
140.0118	140.0118	0.00	Phosphoethanolamine	$C_2H_8NO_4P$	$[M-H]^-$
143.1074	143.1078	−2.47	Caprylic acid	$C_8H_{16}O_2$	$[M-H]^-$
144.0299	144.0302	−2.24	2-Keto-glutaramic acid	$C_5H_7NO_4$	$[M-H]^-$
145.0144	145.0142	1.38	Oxoglutaric acid	$C_5H_6O_5$	$[M-H]^-$
145.0505	145.0506	−0.91	Adipic acid	$C_6H_{10}O_4$	$[M-H]^-$
145.0620	145.0619	0.69	Glutamine	$C_5H_{10}N_2O_3$	$[M-H]^-$
146.0459	146.0459	0.00	Glutamic acid	$C_5H_9NO_4$	$[M-H]^-$
147.0295	147.0299	−2.70	3-Hydroxyglutaric acid	$C_5H_8O_5$	$[M-H]^-$
147.0659	147.0663	−2.91	4,5-Dihydroxyhexanoic acid	$C_6H_{12}O_4$	$[M-H]^-$

Experimental *m*/*z*	Theoretical *m*/*z*	Error (ppm)	Putative assignment	Formula	Ion type*
151.0264	151.0261	1.99	Xanthine	$C_5H_4N_4O_2$	$[M-H]^-$
152.0019	152.0023	−2.78	Cysteine sulfinic acid	$C_3H_7NO_4S$	$[M-H]^-$
154.0622	154.0622	0.00	Histidine	$C_6H_9N_3O_2$	$[M-H]^-$
156.0300	156.0302	−1.39	2-Aminomuconic acid	$C_6H_7NO_4$	$[M-H]^-$
157.0866	157.0870	−2.78	4-Oxooctanoic acid	$C_8H_{14}O_3$	$[M-H]^-$
157.1230	157.1234	−2.56	Pelargonic acid	$C_9H_{18}O_2$	$[M-H]^-$
160.0612	160.0615	−1.76	2-Aminoadipic acid	$C_6H_{11}NO_4$	$[M-H]^-$
161.0451	161.0455	−2.78	3-Hydroxymethylglutaric acid	$C_6H_{10}O_5$	$[M-H]^-$
164.0714	164.0717	−1.84	Phenylalanine	$C_9H_{11}NO_2$	$[M-H]^-$
166.9756	166.9751	2.99	Phosphoenolpyruvic acid	$C_3H_5O_6P$	$[M-H]^-$
167.0212	167.0211	0.60	Uric acid	$C_5H_4N_4O_3$	$[M-H]^-$
168.9908	168.9907	0.59	Glyceraldehyde 3-phosphate	$C_3H_7O_6P$	$[M-H]^-$
171.0066	171.0064	1.17	Glycerol 3-phosphate	$C_3H_9O_6P$	$[M-H]^-$
171.1387	171.1391	−2.06	Capric acid	$C_{10}H_{20}O_2$	$[M-H]^-$
173.0096	173.0092	2.31	Aconitic acid	$C_6H_6O_6$	$[M-H]^-$
174.0405	174.0408	−1.70	*N*-acetyl-aspartic acid	$C_6H_9NO_5$	$[M-H]^-$
175.0249	175.0248	0.57	Ascorbic acid	$C_6H_8O_6$	$[M-H]^-$
176.9356	176.9360	−2.13	Pyrophosphate	$H_4O_7P_2$	$[M-H]^-$
181.0503	181.0506	−1.83	Hydroxyphenyllactic acid	$C_9H_{10}O_4$	$[M-H]^-$
182.0455	182.0459	−2.09	4-Pyridoxic acid	$C_8H_9NO_4$	$[M-H]^-$
184.9852	184.9857	−2.50	Phosphoglyceric acid	$C_3H_7O_7P$	$[M-H]^-$
185.1544	185.1547	−1.64	Undecanoic acid	$C_{11}H_{22}O_2$	$[M-H]^-$
187.1338	187.1340	−0.90	3-Hydroxycapric acid	$C_{10}H_{20}O_3$	$[M-H]^-$
188.0562	188.0564	−1.15	Glutarylglycine	$C_7H_{11}NO_5$	$[M-H]^-$
191.0200	191.0197	1.57	Citric/Isocitric acid	$C_6H_8O_7$	$[M-H]^-$
195.0513	195.0510	1.54	Gulonic acid	$C_6H_{12}O_7$	$[M-H]^-$
197.1551	197.1547	2.03	FA 12:1	$C_{12}H_{22}O_2$	$[M-H]^-$
199.1702	199.1704	−0.77	Dodecanoic acid	$C_{12}H_{24}O_2$	$[M-H]^-$
203.0822	203.0826	−1.97	Tryptophan	$C_{11}H_{12}N_2O_2$	$[M-H]^-$
207.0510	207.0519	−4.32	Dihydrolipoate	$C_8H_{16}O_2S_2$	$[M-H]^-$
214.0489	214.0486	1.40	Glycerylphosphoryl ethanolamine	$C_5H_{14}NO_6P$	$[M-H]^-$
215.0332	215.0328	1.86	Glucose	$C_6H_{12}O_6$	$[M+Cl]^-$
215.1284	215.1289	−2.38	Undecanedioic acid	$C_{11}H_{20}O_4$	$[M-H]^-$
218.1032	218.1034	−0.90	Pantothenic acid	$C_9H_{17}NO_5$	$[M-H]^-$
227.2021	227.2017	1.76	FA 14:0	$C_{14}H_{28}O_2$	$[M-H]^-$
229.0124	229.0119	2.18	Ribose 5-phosphate	$C_5H_{11}O_8P$	$[M-H]^-$
241.2172	241.2173	−0.43	Pentadecanoic acid	$C_{15}H_{30}O_2$	$[M-H]^-$
242.0793	242.0782	4.36	Cytidine	$C_9H_{13}N_3O_5$	$[M-H]^-$
243.0273	243.0275	−0.93	Beta-L-fucose 1-phosphate	$C_6H_{13}O_8P$	$[M-H]^-$

Experimental *m*/*z*	Theoretical *m*/*z*	Error (ppm)	Putative assignment	Formula	Ion type*
243.0628	243.0623	2.06	Uridine	$C_9H_{12}N_2O_6$	$[M-H]^-$
253.2180	253.2173	2.76	FA 16:1	$C_{16}H_{30}O_2$	$[M-H]^-$
255.2336	255.2330	2.35	FA 16:0	$C_{16}H_{32}O_2$	$[M-H]^-$
259.0231	259.0224	2.70	Glucose 3-phosphate	$C_6H_{13}O_9P$	$[M-H]^-$
267.0742	267.0735	2.62	Inosine	$C_{10}H_{12}N_4O_5$	$[M-H]^-$
271.2277	271.2279	−0.62	Hydroxyhexadecanoic acid	$C_{16}H_{32}O_3$	$[M-H]^-$
275.0887	275.0885	0.78	Glutamylglutamic acid	$C_{10}H_{16}N_2O_7$	$[M-H]^-$
277.2181	277.2173	2.89	FA 18:3	$C_{18}H_{30}O_2$	$[M-H]^-$
279.2338	279.2330	2.79	FA 18:2	$C_{18}H_{32}O_2$	$[M-H]^-$
281.2493	281.2486	2.49	FA 18:1	$C_{18}H_{34}O_2$	$[M-H]^-$
282.0852	282.0844	2.84	Guanosine	$C_{10}H_{13}N_5O_5$	$[M-H]^-$
283.2648	283.2643	1.77	FA 18:0	$C_{18}H_{36}O_2$	$[M-H]^-$
295.2277	295.2279	−0.57	Hydroxyoctadecadienoic acid	$C_{18}H_{32}O_3$	$[M-H]^-$
300.0401	300.0395	2.00	*N*-Acetylgalactosamine 6-sulfate	$C_8H_{15}NO_9S$	$[M-H]^-$
301.2182	301.2173	2.98	FA 20:5	$C_{20}H_{30}O_2$	$[M-H]^-$
303.0832	303.0834	−0.62	*N*-Acetylaspartylglutamic acid	$C_{11}H_{16}N_2O_8$	$[M-H]^-$
303.2336	303.2330	1.98	FA 20:4	$C_{20}H_{32}O_2$	$[M-H]^-$
306.0773	306.0765	2.61	Glutathione	$C_{10}H_{17}N_3O_6S$	$[M-H]^-$
307.2650	307.2643	2.28	FA 20:2	$C_{20}H_{36}O_2$	$[M-H]^-$
308.0986	308.0987	−0.32	*N*-Acetylneuraminic acid	$C_{11}H_{19}NO_9$	$[M-H]^-$
309.2807	309.2799	2.59	FA 20:1	$C_{20}H_{38}O_2$	$[M-H]^-$
311.2963	311.2956	2.25	FA 20:0	$C_{20}H_{40}O_2$	$[M-H]^-$
313.2383	313.2384	−0.42	FA 18:1;O2	$C_{18}H_{34}O_4$	$[M-H]^-$
322.0456	322.0446	3.11	Cytidine 3'-monophosphate	$C_9H_{14}N_3O_8P$	$[M-H]^-$
323.0294	323.0286	2.48	Uridine 3'-monophosphate	$C_9H_{13}N_2O_9P$	$[M-H]^-$
327.2338	327.2330	2.44	FA 22:6	$C_{22}H_{32}O_2$	$[M-H]^-$
329.2498	329.2486	3.64	FA 22:5	$C_{22}H_{34}O_2$	$[M-H]^-$
331.2650	331.2643	2.11	FA 22:4	$C_{22}H_{36}O_2$	$[M-H]^-$
333.2802	333.2799	0.89	FA 22:3	$C_{22}H_{38}O_2$	$[M-H]^-$
335.2227	335.2228	−0.25	Hepoxilin B3	$C_{20}H_{32}O_4$	$[M-H]^-$
337.3112	337.3112	−0.01	FA 22:1	$C_{22}H_{42}O_2$	$[M-H]^-$
338.9898	338.9888	2.95	Fructose 1,6-bisphosphate	$C_6H_{14}O_{12}P_2$	$[M-H]^-$
343.2280	343.2279	0.38	FA 22:6;O	$C_{22}H_{32}O_3$	$[M-H]^-$
346.0568	346.0558	2.89	Adenosine 5’-monophosphate	$C_{10}H_{14}N_5O_7P$	$[M-H]^-$
347.0409	347.0398	3.17	Inosinic acid	$C_{10}H_{13}N_4O_8P$	$[M-H]^-$
351.2181	351.2177	1.14	Prostaglandin D2	$C_{20}H_{32}O_5$	$[M-H]^-$
353.2335	353.2333	0.43	Prostaglandin F2α	$C_{20}H_{34}O_5$	$[M-H]^-$
359.2955	359.2956	−0.15	FA 24:4	$C_{24}H_{40}O_2$	$[M-H]^-$
362.0518	362.0507	3.04	Guanosine 3'-monophosphate	$C_{10}H_{14}N_5O_8P$	$[M-H]^-$

**Experimental *m*/*z***	**Theoretical *m*/*z***	**Error (ppm)**	**Putative assignment**	**Formula**	**Ion type***
365.3424	365.3425	−0.28	FA 24:1	$C_{24}H_{46}O_2$	$[M-H]^-$
402.9960	402.9949	2.73	Uridine 5'-diphosphate	$C_9H_{14}N_2O_{12}P_2$	$[M-H]^-$
426.0233	426.0221	2.82	Adenosine diphosphate	$C_{10}H_{15}N_5O_{10}P_2$	$[M-H]^-$
442.0184	442.0171	2.94	Guanosine diphosphate	$C_{10}H_{15}N_5O_{11}P_2$	$[M-H]^-$
445.0544	445.0531	2.92	CDP-ethanolamine	$C_{11}H_{20}N_4O_{11}P_2$	$[M-H]^-$
465.3053	465.3044	1.93	Cholesterol sulfate	$C_{27}H_{46}O_4S$	$[M-H]^-$

* $[M+Cl]^-$ ions were manually validated by the presence and abundance ratio of their ^{37}Cl isotopologue peaks.

References

1. D.-Q. Jin, Y. Zhu, and Q. Fang, "Swan Probe: A Nanoliter-Scale and High-Throughput Sampling Interface for Coupling Electrospray Ionization Mass Spectrometry with Microfluidic Droplet Array and Multiwell Plate," *Analytical Chemistry 86*, no. 21 (2014): 10796–10803, https://doi.org/10.1021/ac503014k.
2. X. Li, H. Hu, M. Yang, and J. Laskin, "A Low-Cost, High-Resolution Thermoplastic Microfluidic Probe for Mass Spectrometry Imaging of Biological Tissue Samples," *Analytical Chemistry 97*, no. 6 (2025): 3207–3212, https://doi.org/10.1021/acs.analchem.4c06087.
3. L. Jiang, R. T. Hilger, and J. Laskin, "Hardware and Software Solutions for Implementing Nanospray Desorption Electrospray Ionization (nano-DESI) Sources on Commercial Mass Spectrometers," *Journal of Mass Spectrometry 59*, no. 7 (2024): e5065, https://doi.org/10.1002/jms.5065.
4. M. Yang, D. Unsihuay, H. Hu, et al., "Nano-DESI Mass Spectrometry Imaging of Proteoforms in Biological Tissues with High Spatial Resolution," *Analytical Chemistry 95*, no. 12 (2023): 5214–5222, https://doi.org/10.1021/acs.analchem.2c04795.
5. R. Yin, K. E. Burnum-Johnson, X. Sun, S. K. Dey, and J. Laskin, "High Spatial Resolution Imaging of Biological Tissues Using Nanospray Desorption Electrospray Ionization Mass Spectrometry," *Nature Protocols 14*, no. 12 (2019): 3445–3470, https://doi.org/10.1038/s41596-019-0237-4.
6. X. Li, H. Hu, and J. Laskin, "High-Resolution Integrated Microfluidic Probe for Mass Spectrometry Imaging of Biological Tissues," *Analytica Chimica Acta 1279* (2023): 341830, https://doi.org/10.1016/j.aca.2023.341830.
7. X. Li, R. Yin, H. Hu, et al., "An Integrated Microfluidic Probe for Mass Spectrometry Imaging of Biological Samples," *Angewandte Chemie International Edition 59*, no. 50 (2020): 22388–22391, https://doi.org/10.1002/anie.202006531.
8. X. Li, H. Hu, R. Yin, et al., "High-Throughput Nano-DESI Mass Spectrometry Imaging of Biological Tissues Using an Integrated Microfluidic Probe," *Analytical Chemistry 94*, no. 27 (2022): 9690–9696, https://doi.org/10.1021/acs.analchem.2c01093.
9. L.-X. Jiang, M. Polack, X. Li, M. Yang, D. Belder, and J. Laskin, "A Monolithic Microfluidic Probe for Ambient Mass Spectrometry Imaging of Biological Tissues," *Lab on a Chip 23*, no. 21 (2023): 4664–4673, https://doi.org/10.1039/D3LC00637A.
10. R. Dong, A. H. Choi, X. Li, X. Tang, L.-X. Jiang, and J. Laskin, "Fabrication of Thermoplastic Microfluidic Probes for Nanospray Desorption Electrospray Ionization Mass Spectrometry," *International Journal of Mass Spectrometry 528* (2026): 117672, https://doi.org/10.1016/j.ijms.2026.117672.
11. L.-X. Jiang, X. Li, M. Polack, D. Belder, and J. Laskin, "High-Spatial-Resolution Mass Spectrometry Imaging of Biological Tissues Using a Microfluidic Probe," *Nature Protocols 21*, no. 1 (2026): 18–36, https://doi.org/10.1038/s41596-025-01188-y.
12. O. J. Hale, T. R. Wells, R. J. Mead, and H. J. Cooper, "Mass Spectrometry Imaging of SOD1 Protein-Metal Complexes in SOD1G93A Transgenic Mice Implicates Demetalation with Pathology," *Nature Communications 15*, no. 1 (2024): 6518, https://doi.org/10.1038/s41467-024-50514-7.
